# Development of a multi-feature-combined model: proof-of-concept with application to local failure prediction of post-SBRT or surgery early-stage NSCLC patients

**DOI:** 10.3389/fonc.2023.1185771

**Published:** 2023-09-13

**Authors:** Zhenyu Yang, Chunhao Wang, Yuqi Wang, Kyle J. Lafata, Haozhao Zhang, Bradley G. Ackerson, Christopher Kelsey, Betty Tong, Fang-Fang Yin

**Affiliations:** ^1^ Department of Radiation Oncology, Duke University, Durham, NC, United States; ^2^ Medical Physics Graduate Program, Duke Kunshan University, Kunshan, Jiangsu, China; ^3^ Medical Physics Graduate Program, Duke University, Durham, NC, United States; ^4^ Department of Electrical and Computer Engineering, Duke University, Durham, NC, United States; ^5^ Department of Radiology, Duke University, Durham, NC, United States; ^6^ Department of Surgery, Duke University, Durham, NC, United States

**Keywords:** early-stage lung NSCLC, local failure, surgery, SBRT, machine learning (ML), deep learning

## Abstract

**Objective:**

To develop a Multi-Feature-Combined (MFC) model for proof-of-concept in predicting local failure (LR) in NSCLC patients after surgery or SBRT using pre-treatment CT images. This MFC model combines handcrafted radiomic features, deep radiomic features, and patient demographic information in an integrated machine learning workflow.

**Methods:**

The MFC model comprised three key steps. (1) Extraction of 92 handcrafted radiomic features from the GTV segmented on pre-treatment CT images. (2) Extraction of 512 deep radiomic features from pre-trained U-Net encoder. (3) The extracted handcrafted radiomic features, deep radiomic features, along with 4 patient demographic information (i.e., gender, age, tumor volume, and Charlson comorbidity index), were concatenated as a multi-dimensional input to the classifiers for LR prediction. Two NSCLC patient cohorts from our institution were investigated: (1) the surgery cohort includes 83 patients with segmentectomy or wedge resection (7 LR), and (2) the SBRT cohort includes 84 patients with lung SBRT (9 LR). The MFC model was developed and evaluated independently for both cohorts, and was subsequently compared against the prediction models based on only handcrafted radiomic features (R models), patient demographic information (PI models), and deep learning modeling (DL models). ROC with AUC was adopted to evaluate model performance with leave-one-out cross-validation (LOOCV) and 100-fold Monte Carlo random validation (MCRV). The *t*-test was performed to identify the statistically significant differences.

**Results:**

In LOOCV, the AUC range (surgery/SBRT) of the MFC model was 0.858-0.895/0.868-0.913, which was higher than the three other models: 0.356-0.480/0.322-0.650 for PI models, 0.559-0.618/0.639-0.682 for R models, and 0.809/0.843 for DL models. In 100-fold MCRV, the MFC model again showed the highest AUC results (surgery/SBRT): 0.742-0.825/0.888-0.920, which were significantly higher than PI models: 0.464-0.564/0.538-0.628, R models: 0.557-0.652/0.551-0.732, and DL models: 0.702/0.791.

**Conclusion:**

We successfully developed an MFC model that combines feature information from multiple sources for proof-of-concept prediction of LR in patients with surgical and SBRT early-stage NSCLC. Initial results suggested that incorporating pre-treatment patient information from multiple sources improves the ability to predict the risk of local failure.

## 1 Introduction

Lung cancer is the leading cause of cancer death worldwide (1, 2). The global cancer statistics show that there were 2.2 million cancer diagnoses and 1.8 million deaths in 2020, with lung cancer accounting for approximately one in ten (11.4%) cancer diagnoses and one in five (18.0%) cancer deaths (1). Non-small cell lung cancer (NSCLC) accounts for more than 80% of all lung cancers (3). With the growing acceptance of computed tomography (CT) screening, more than one-fifth of NSCLC patients are diagnosed at an early stage (4). Surgery is the current standard-of-care treatment modality for early-stage NSCLC (3, 5, 6), with a reported 5-year overall survival (OS) rate of 73% (7, 8). Despite the promising OS rates that have been shown with surgery, only approximately 70% of early-stage NSCLC patients receive surgical treatment (9, 10). The remaining approximately 30% of patients are considered surgically unsuitable due to severe comorbidities, such as cardiac dysfunction or poor lung function; in addition, some patients may decline surgery for personal reasons (10). Recently, stereotactic body radiation therapy (SBRT) has been reported as a promising alternative treatment option for early-stage NSCLC (11, 12). Compared to conventional radiation therapy, SBRT employs a substantially higher dose per treatment session in the target region with a sharp dose fall-off gradient, resulting in improved local control rates and decreased toxicity to surrounding structures (12, 13). A recent CHISEL phase III randomized controlled trial has confirmed the effectiveness of SBRT (14), and the American Society for Radiation Oncology (ASTRO) has developed evidence-based guidelines for SBRT treatment in early-stage NSCLC patients (15). Several retrospective studies have reported that the outcome of SBRT has the potential to be comparable to surgery (16–18).

With various treatment techniques available, the accurate identification of patients at high risk following SBRT or surgical treatment is desired to improve both risk stratification and potential patient survival (19, 20). The earlier studies included outcome prediction (for both surgery and SBRT treatment) based only on the patient demographic information and tumor information (e.g., TNM staging information) (21, 22); however, limited performance has been reported due to the complexity of the prognostic prediction problem in early-stage NSCLC. Many efforts have since been made toward image-based treatment outcome prediction based on the wide accessibility of pre-treatment CT images (23–25). As a popular image quantification method, radiomic analysis has been successfully demonstrated in several retrospective studies (23, 24). The classic radiomic analysis employs manual feature engineering from domain experts to extract the handcrafted image intensity and texture features (namely, handcrafted radiomic features or engineered radiomic features) from a pre-defined volume-of-interest (VOI), e.g., tumor region. The extracted features serve as potential biomarkers reflecting the underlying pathophysiology, which can be modeled by classic machine learning classifiers (e.g., logistic regression, supporting vector machine, random forest, etc.) to associate with the clinical outcomes (26). Separate pilot studies have shown that the classic radiomic analysis based on the pre-treatment CT has the potential to predict OS (25, 27), disease-free survival (28, 29), metastasis (30), and stratification (31) in early-stage NSCLC following surgical or SBRT treatment. Recently, deep learning-based radiomic analysis, represented by the deep neural network (DNN), has been considered a new approach for image quantification and characterization (32). Driven by advancements in computational hardware, algorithms, and big data, DNN with convolutional operations directly learns high-level abstractions using the paired medical image and outcome ground truth. The data pass through the DNN in a hierarchical and nested fashion without requiring manual feature engineering (33–35). The latent variables linking the input (i.e., medical image) and output spaces (i.e., clinical outcome) are considered potential radiomic features (namely, deep radiomic features) (36). The deep learning methods have also been successfully applied to prognosis prediction in early-stage NSCLC, including OS, metastasis, stratification, etc. (37, 38).

While many classic radiomic- and deep radiomic-based image quantifications have been successfully demonstrated in early-stage NSCLC, the majority of these studies rely only on a single patient cohort (i.e., patients following either surgery or SBRT treatment) for model development. Few studies have yet focused on developing and evaluating a clinical outcome prediction model for both surgery and SBRT patient cohorts. This work aimed to develop a technically novel Multi-Feature-Combined (MFC) model to predict local failure from pre-treatment CT imaging for both early-stage NSCLC surgery and SBRT patients. The technical innovations of the model include (1) performing both classic radiomic analysis and deep learning-based radiomic analysis on pre-treatment CT images of lung surgery and SBRT patient cohorts, and (2) combining the obtained handcrafted radiomic features, deep radiomic features, as well as four patient demographic variables as an integrated workflow for local failure prediction. Additionally, comparison studies were designed to evaluate the prediction performance of MFC against other established methods.

## 2 Materials and methods

### 2.1 Patient data

Two NSCLC patient cohorts from our institution, i.e., the surgery cohort and the SBRT cohort, were investigated in this IRB-approved retrospective study. All patients undergoing sub-lobar resection or SBRT at our institution for stage I NSCLC from 2007 to 2014 were evaluated. All the patients were treated at the discretion of the treating physicians following a multidisciplinary evaluation. For the patients in the surgery cohort, lobectomy was considered based on overall performance status and objective evaluation of pulmonary function. Anatomic sub-mediastinal resection with mediastinal lymph node dissection was preferred when lobectomy was not considered feasible, but wedge resection was performed in patients with co-morbid advanced disease, previous complex resections, or very peripheral lesions. For the patients in the SBRT cohort, 3-dimensional conformal irradiation was the most common treatment technique, with the occasional use of intensity-modulated radiation therapy (IMRT) or volumetric intensity-modulated arc therapy (VMAT) to preserve vital normal tissues. SBRT procedures were delivered in 3-5 fractions of radiation every 48-72 hours. The fractionation schemes utilized were almost exclusively 10Gy × 5, 12-12.5 Gy × 4, or 18-20 Gy × 3.

Patients were excluded based on the following criteria: (1) patients found to have pathologically involved lymph nodes; (2) patients with previous lung cancer or multiple synchronous primary cancers. For the SBRT cohort, patients were further excluded based on the following criteria: (a) no biopsy was performed; (b) inconclusive, non-specific biopsy results; (c) significant chest wall invasion. Based on the above criteria, the obtained dataset includes 83 surgery patients (i.e., surgery cohort) and 84 SBRT patients (i.e., SBRT cohort) (39, 40).

All pre-treatment CT images were acquired under free-breathing conditions for both patient cohorts, and all images were resampled with 1×1×1 mm^3^ isotropic voxel size for the following image analysis. The gross tumor volume (GTV) was identified for both patient cohorts by experienced physicians using the Eclipse™ software (Varian Medical System, Palo Alto, CA) with a similar standard. The lesion sizes and appearances were cross-checked between two patient cohorts to ensure appropriate delineation of lymph vascular structures. Patient demographic information, including gender, age at diagnosis, tumor volume, and Charlson comorbidity index (CCI) (41), were collected to provide complementary patient information. The prescription dose and the fractionation scheme were added to provide additional treatment information to the prediction modeling of SBRT cohorts.

The treatment outcome was subsequently evaluated for both patient cohorts based on the available follow-up CT scans, PET/CT scans, or pathological information. According to national guidelines, most patients were monitored with chest CT every 3-6 months for the first two years, and then annually thereafter (39, 40). PET-CT imaging for surveillance was not routinely prescribed but may be obtained to evaluate suspicious CT findings. In this work, the local failure was studied independently for two patient cohorts: 1) local failure for the surgery cohort refers to recurrence along the surgical suture line based on radiologic interpretation (42); 2) local failure for the SBRT cohort refers to the recurrence at the site of treatment (within the initial planning target volume, PTV) (43). Due to the difficulty in distinguishing local failures after SBRT, two authors (BA and CK) reviewed post-SBRT imaging, particularly PET-CT imaging, and other diagnostic studies to evaluate failure patterns. Based on the above workflow, 7 and 9 local failures were identified for the surgery and SBRT cohorts, respectively. The surgery patient and SBRT patient data included in this study are summarized in **Table 1**. More detailed patient background information can be found in the **Supplementary Materials**.

**Table 1:** Summary of surgery patient and SBRT patient data included in this study.

	Surgery Patient Cohort	SBRT Patient Cohort
**# Subjects, Total**	83	84
**# Subjects, Local Recurrence**	7	9
**Gender**	47% Male, 53% Female	56% Male, 44% Female
**Age**	Ave: 70, Range: 51-88	Ave: 83, Range: 52-100
**Tumor Size (cc)**	Ave: 1.75, Range: 0.6-6	Ave: 2.36, Range: 0.9-4.7
**CT Acquisition Mode**	Helical	Helical
**CT Tube Voltage**	120 kVp	120 kVp
**CT Slice Thickness (# Subjects)**	< 1.25 mm (11) 1.25 mm - 3 mm (3) > 3 mm (69)	< 1.25 mm (2) 1.25 mm - 3 mm (81) > 3 mm (1)
**CT In-plane Resolution (# Subjects)**	< 0.75 mm (30) 0.75 – 1.25 mm (52) >1.25 mm (1)	< 0.75 mm (0) 0.75 – 1.25 mm (68) >1.25 mm (16)

### 2.2 MFC Model design

**Figure 1:**
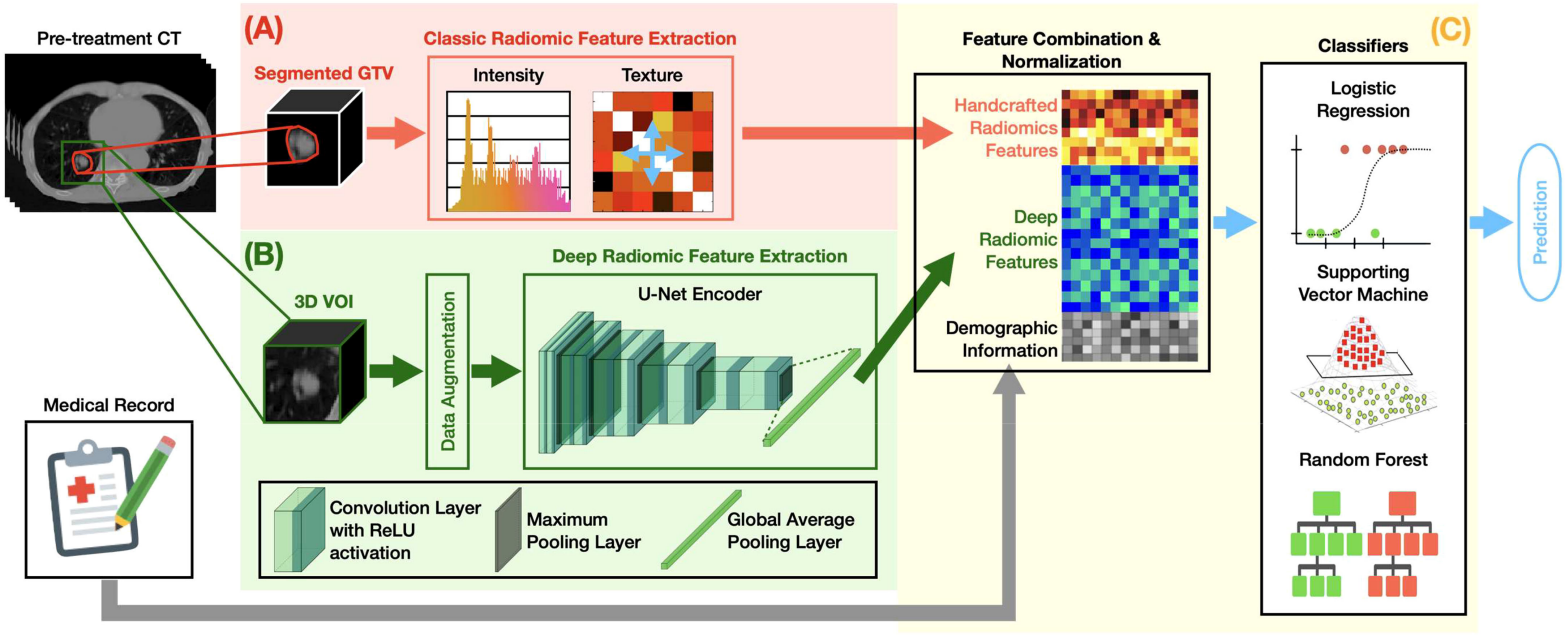
The overall design of the MFC model, which comprised three key steps: **(A)** handcrafted radiomic feature extraction, **(B)** deep radiomic feature extraction, and **(C)** machine learning implementation for outcome prediction.

**Figure 1** summarizes the overall design of our MFC model. The MFC comprised three key steps: (A) handcrafted radiomic feature extraction, (B) deep radiomic feature extraction, and (C) machine learning implementation for outcome prediction.

#### 2.2.1 Handcrafted radiomic feature extraction

**Figure 1A** shows the classic radiomic feature extraction workflow. The 3D GTV volumes were first segmented from the pre-treatment lung CT images for both surgery and SBRT patient cohorts, from which a total of 105 handcrafted radiomic features were extracted. These features can be grouped into 6 categories based on their different joint-probability functions: 13 shape-based morphological features, 18 intensity-based features, 20 histogram-based features, 22 gray-level co-occurrence matrix (GLCOM)-based features, 16 gray-level run length matrix (GLRLM)-based features, and 16 gray-level size zone matrix (GLSZM)-based features. A complete list of the extracted handcrafted features can be found in the **Supplementary Materials**. The obtained 105-dimensional feature vector served as a computational biomarker capturing the overall morphological and texture characteristics within the GTV. The fixed bin number (= 32) image discretization was employed for calculating second-order features (i.e., histogram-based, GLCOM-based, GLRLM-based, and GLSZM-based features) (44, 45). The 13 shape-based features and 18 intensity-based features were extracted based on the original segmented 3D GTV volumes (46). All radiomics analyses were carried out based on our in-house developed radiomics calculation platform using MATLAB (The MathWorks, Natick, MA) (35). The entire feature extraction workflow has been fully calibrated against the image biomarker standardization initiative (IBSI) (46, 47).

#### 2.2.2 Deep radiomic feature extraction

**Figure 1B** demonstrates the deep radiomic feature extraction workflow. A pre-trained U-Net (48) encoder [namely, Generic Autodidactic Models or Genesis model (49)] was adopted as a transfer learning strategy to deal with the relatively small dataset. The Genesis model was trained to learn generic anatomical representation from medical images via a unified self-supervised learning framework, and has been shown to produce state-of-the-art results in image segmentation and classification tasks (49). The U-Net encoder consists of five repeated convolutional blocks: each block contains two convolutional layers with rectified linear unit activation followed by a max pooling operation. In this process, the spatial dimension decreases while the feature information increases (from 1 channel grayscale image to a 512-channel feature representation). A global average pooling layer was followed to average each channel of the feature representation. By initializing the U-Net encoder with the pre-trained weight from the Genesis model, the original image can be encoded into a 512-dimensional feature vector as deep features.

The 3D 8×8×8 cm^3^ VOI fully containing the GTV in its center was utilized as the deep learning model’s input. The 3D-based data augmentation (including rotation, scaling, flipping, and adding noise) was implemented to enhance training sample data utilization and prevent potential overfitting due to imbalanced outcome distribution. All the deep learning design was implemented under a Python environment with Tensorflow 2.5.0.

#### 2.2.3 Local failure prediction

**Figure 1C** summarizes the machine learning implementation. The extracted 105 handcrafted radiomic features, 512 deep radiomic features, and patient demographic information (and treatment information for the SBRT cohort) were concatenated as a multi-dimensional input for the subsequent modeling. The oversampling with Gaussian noise was adopted as the data augmentation technique for the handcrafted radiomics features to match the data dimension of the deep radiomic features. The z-score normalization was performed to normalize the combined feature space with respect to each feature’s mean value in the training set. Three classic machine learning classifiers — logistic regression (LR), support vector machine (SVM), and random forest (RF) — were employed to investigate the multivariate association between the combined features and clinical endpoints.

To reduce the potential overfitting during the model training, the multicollinearity assessment was performed to remove the redundant features (i.e., the features that are highly correlated to each other). For each feature, Pearson correlation coefficients (*r*) were calculated against the rest features, and the feature pairs with $\bar{r}$ > 0.95 were considered highly correlated (50, 51). The highly correlated features were classified as a feature subset, and the univariate statistical tests were employed to obtain one dependent variable within each feature subset: the feature with the smallest *p*-value compared against the final local failure was considered independent. The obtained features were considered as independent inputs to the subsequent modeling and the interaction between variables thus are not considered in subsequent modeling.

The developed MFC model was independently trained and evaluated for surgery patient and SBRT patient cohorts. The following settings were employed for the classifiers to optimize the model performance:

1. For the LR classifier, L2 regulation with a limited-memory BFGS solver was employed.
2. For the SVM classifier, the radial basis function kernel was selected with a tolerance equal to 10^-3^.
3. For the RF classifier, the number of trees and the maximum tree depth were decided by observing the performance between the test and training set to pick up the maximum depth before over-fitting.

Two validation methods were employed to objectively evaluate the prediction performance: leave-one-out cross-validation (LOOCV) (52) and 100-fold Monte Carlo random validation (MCRV) (53). For LOOCV, each sample is used once as a test set, and the remaining samples comprise the training set. The LOOCV method has been proven to provide a less biased model performance measurement, especially for small datasets. For 100-fold MCRV, the model was trained independently with 100 versions of random 70%-30% training test assignments (i.e., surgery: 53 non-local failure and 5 local failure samples for training; SBRT: 52 non-local failure and 6 local failure samples for training). Mean receiver operating characteristic (ROC) with the area under the curve (AUC) and confusion metrics (i.e., sensitivity, specificity, and accuracy) were summarized to quantify the prediction performance for both validation methods.

### 2.3 Comparison Studies

**Figure 2:**
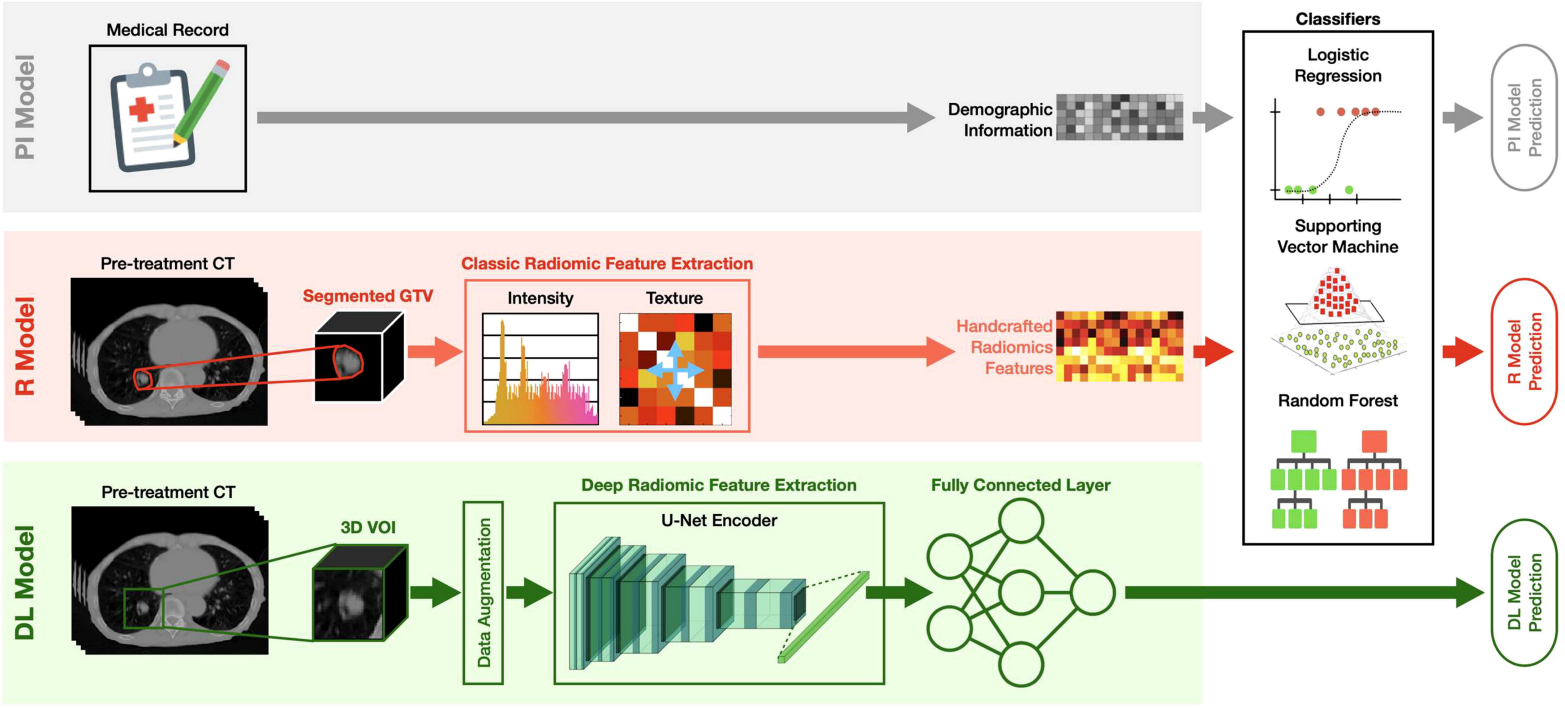
The design of the PI model, R model, and DL model. PI model: the prediction model based on LR/SVM/RF classifiers using only 4 patient demographic information (plus 2 more treatment information for the SBRT cohort) as inputs; R model: the prediction model based on LR/SVM/RF classifiers using only 105 handcrafted radiomic features as inputs; DL model: deep learning prediction model based on the pre-trained U-Net encoder (i.e., Genesis model). Another fully connected (FC) layer with sigmoid activation was added to obtain the final binary diagnosis label directly.

As summarized in **Figure 2**, the developed MFC model was subsequently compared to three additional prediction models:

1. PI model: the prediction model based on LR/SVM/RF classifiers using only 4 patient demographic information (plus 2 more treatment information for the SBRT cohort) as inputs. The model settings for the three classifiers are kept the same as the MFC model.
2. R model: the prediction model based on LR/SVM/RF classifiers using only 105 handcrafted radiomic features as inputs. The model settings for the three classifiers are kept the same as the MFC model. Oversampling was utilized during the model training to match the data utilization in the MFC model.
3. DL model: deep learning prediction model based on the pre-trained U-Net encoder (i.e., Genesis model). Another fully connected layer (FCL) with sigmoid activation was trained to obtain the final binary diagnosis label directly. The Adam optimizer with binary cross-entropy loss function was employed during the model training. The other training settings, including transfer learning strategies and data augmentation, were kept the same as the proposed MFC model (as shown in **Figure 1B**).

All the comparison models were trained and evaluated for two patient cohorts independently, and two cross-validation methods (i.e., LOOCV and 100-fold MCRV) were kept the same as the MFC model. Multicollinearity assessment was also performed to remove the redundant features for the PI models and R models. The confusion metrics (i.e., sensitivity, specificity, accuracy) and AUC results from ROC were calculated. In the 100-fold MRCV, the AUC results of different folds were compared to the MFC model using Student’s *t*-test, with a significance level of 0.05 when applicable.

## 3 Results

**Figure 3:**
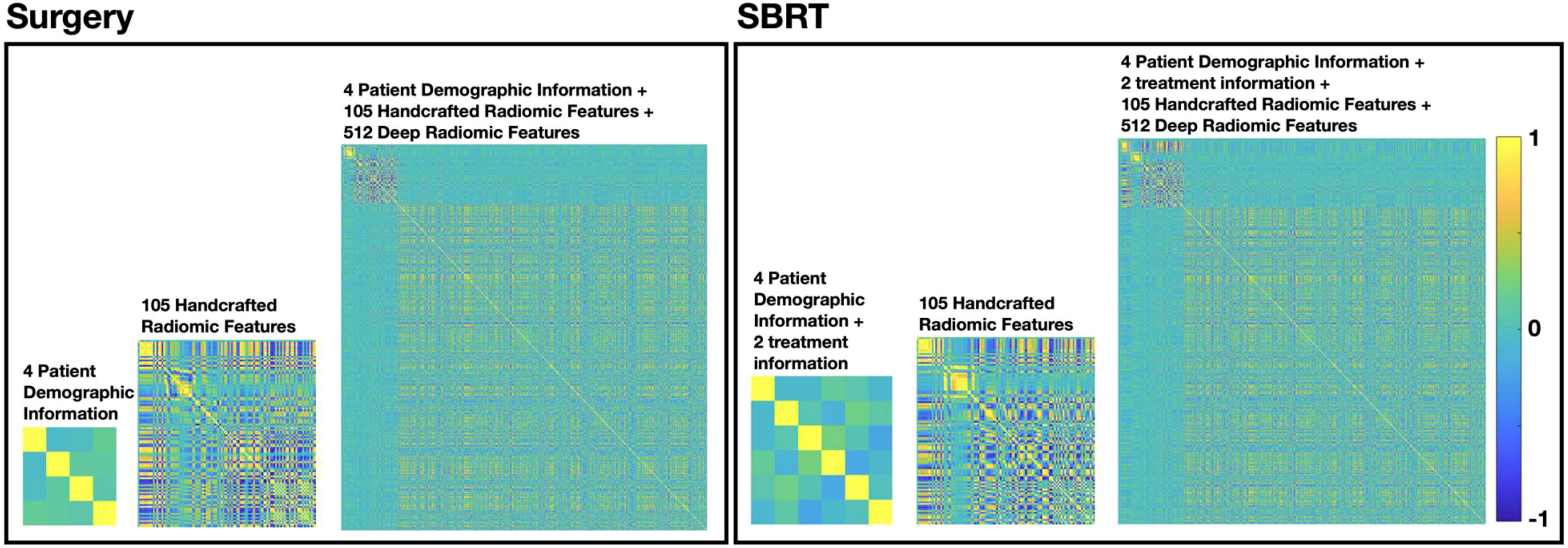
The multicollinearity assessment results for each feature pair in surgery and SBRT patient cohorts, respectively.

**Figure 3** summarized the multicollinearity assessment results for each feature pair in both patient cohorts. For the PI model, all 4 patient demographic information (and 2 more treatment information for the SBRT model) were identified as independent variables for both cohorts. For the R model, 61 radiomics features (including 8 shape features, 13 intensity features, 17 histogram-based features, 15 GLCOM-based features, 4 GLRLM-based features, and 4 GLSZM-based features) were found to be independent in the surgery cohort, while 55 radiomics features (including 9 shape features, 9 intensity features, 15 histogram-based features, 15 GLCOM-based features, 2 GLRLM-based features, and 5 GLSZM-based features) were found to be independent in the SBRT cohort. The identified independent radiomics features in both patient cohorts showed similar feature types and number distributions. In the MFC model, all the above features were kept independent, and 491 and 506 independent deep features were additionally identified for the surgery and SBRT cohort, respectively.

**Figure 4:**
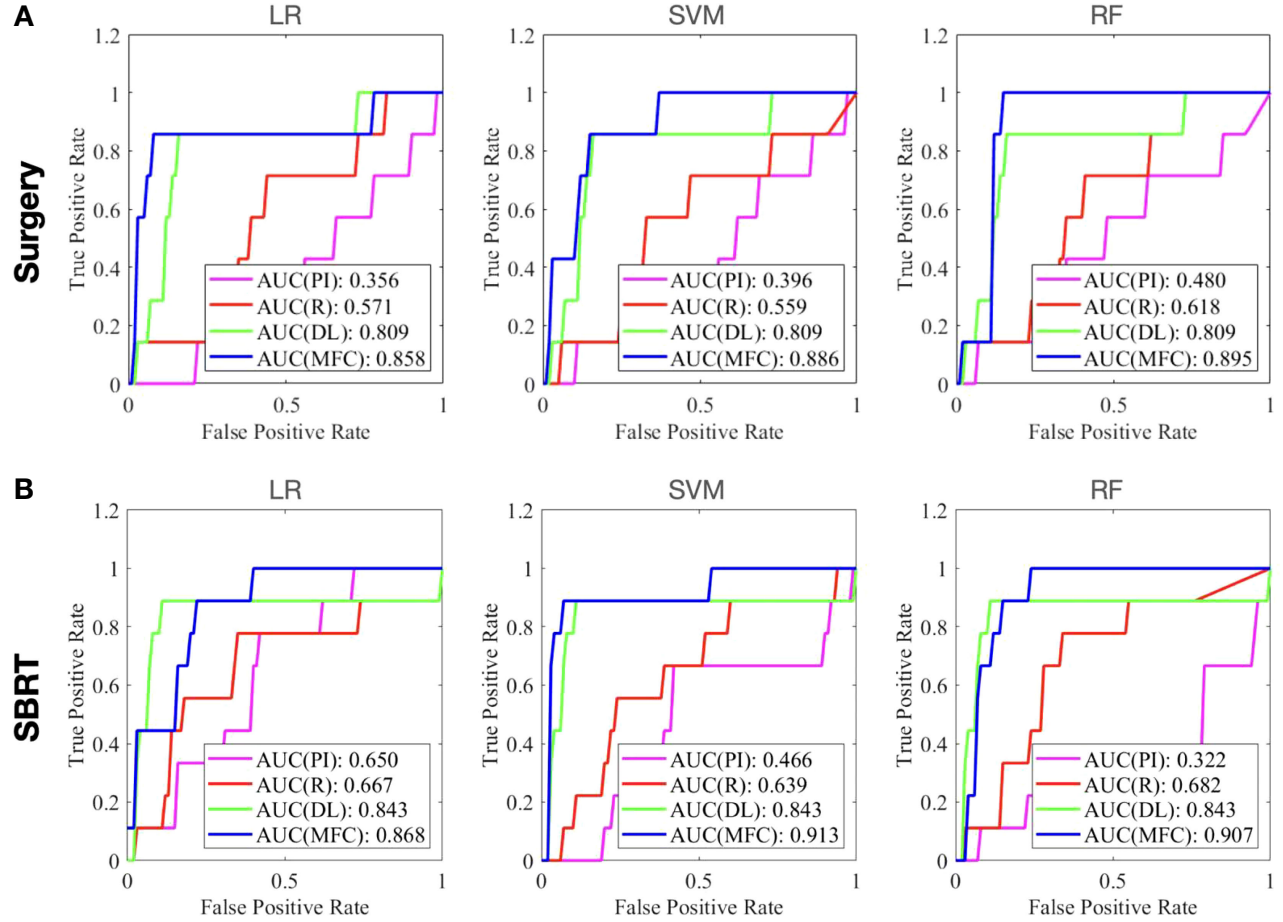
The ROC results of LOOCV from the MFC model (blue lines), R model (red lines), PI model (pink lines), and DL model (green lines) for **(A)** surgery patient cohort and **(B)** SBRT patient cohort, respectively.

**Figure 5:**
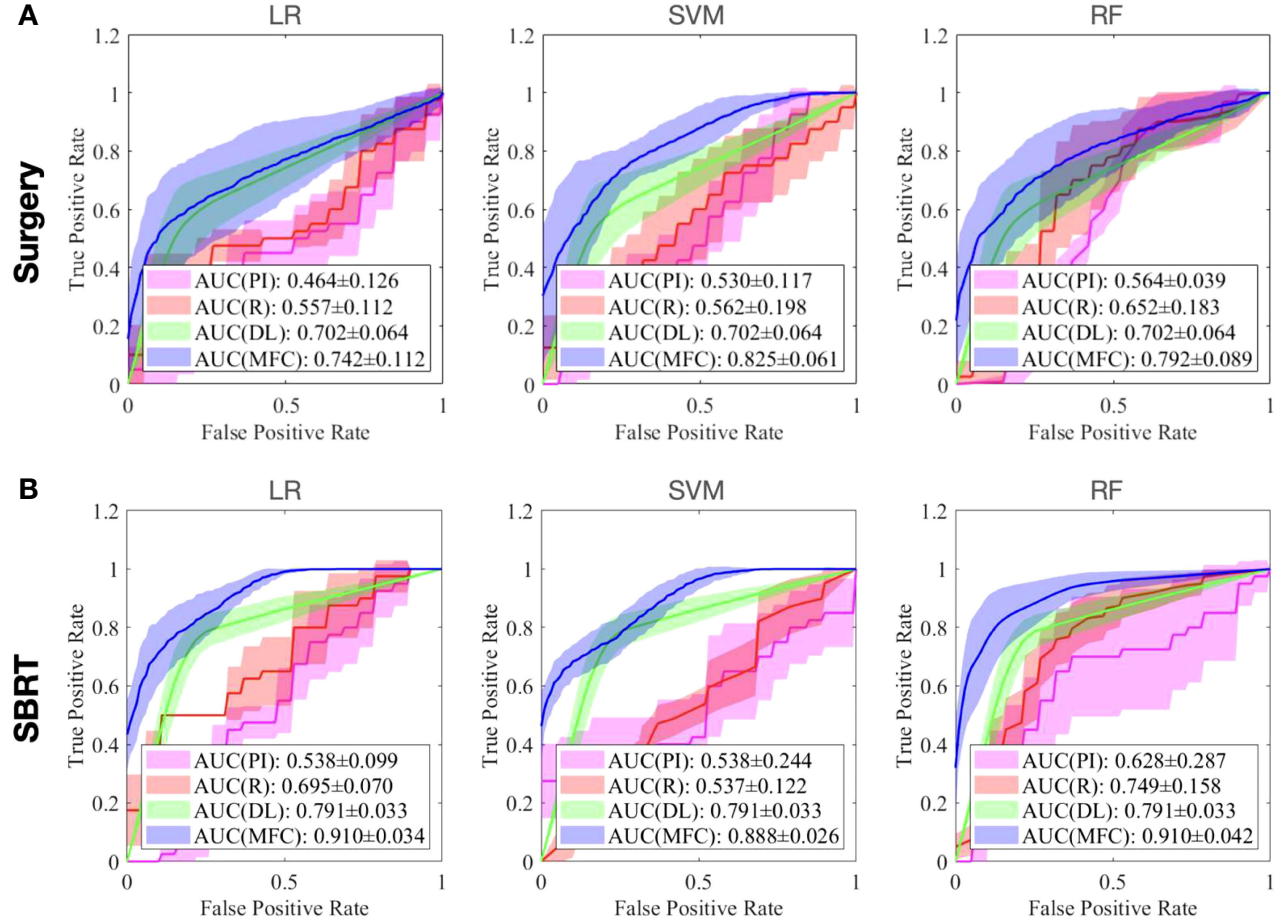
The ROC results of 100-fold MCRV from the MFC model (blue lines), R model (red lines), PI model (pink lines), and DL model (green lines) for **(A)** surgery patient cohort and **(B)** SBRT patient cohort, respectively. The colored shade areas represent the variance over 100 folds.

**Figure 4** compared the ROC results of LOOCV from the MFC model (blue lines), R model (red lines), PI model (pink lines), and DL model (green lines) for (A) surgery patient cohort and (B) SBRT patient cohort, respectively. The ROC results of 100-fold MCRV were shown in **Figures 5A, B** for the surgery and SBRT cohorts, respectively. The colored shade areas represented the variance over 100 different folds. **Tables 2** and **3** summarized the corresponding quantitative AUC, sensitivity, specificity, and accuracy results for the two validation methods, respectively. **Table 4** showed the *p*-value results of the AUC comparison results, where marker “*” indicated the statistical significance (i.e., *p*-value<0.05).

**Table 2:** AUC, sensitivity, specificity, and accuracy results of PI, R, DL, and MFC models for surgery and SBRT patient cohorts in the LOOCV method.

		PI Model			R Model			DL Model	MFC Model		
		LR	SVM	RF	LR	SVM	RF	FCL	LR	SVM	RF
LOOCV (Surgery)	**AUC**	0.356	0.396	0.480	0.571	0.559	0.618	0.809	**0.858**	**0.886**	**0.895**
	**Sensitivity**	0.143	0.143	0.143	0.286	0.286	0.286	0.428	0.571	0.429	0.857
	**Specificity**	0.671	0.684	0.895	0.671	0.711	0.671	0.934	0.961	0.947	0.882
	**Accuracy**	0.627	0.639	0.831	0.639	0.675	0.637	0.892	0.928	0.904	0.880
LOOCV (SBRT)	**AUC**	0.650	0.466	0.322	0.667	0.639	0.682	0.843	**0.868**	**0.913**	**0.907**
	**Sensitivity**	0.333	0.000	0.000	0.556	0.222	0.111	0.556	0.444	0.889	0.778
	**Specificity**	0.693	0.800	0.933	0.813	0.813	0.907	0.933	0.907	0.933	0.867
	**Accuracy**	0.655	0.726	0.833	0.786	0.750	0.821	0.893	0.857	0.929	0.857

Bold values represent the best statistically significant results.

**Table 3:** AUC, sensitivity, specificity, and accuracy results of PI, R, DL, and MFC models for surgery and SBRT patient cohorts in the 100-fold MCRV method.

		PI Model			R Model			DL Model	MFC Model		
		LR	SVM	RF	LR	SVM	RF	FCL	LR	SVM	RF
MCRV (Surgery)	**AUC**	0.464 ± 0.126	0.530 ± 0.117	0.564 ± 0.039	0.557 ± 0.112	0.562 ± 0.112	0.652 ± 0.183	0.702 ± 0.064	**0.742 ± 0.112**	**0.825 ± 0.061**	**0.792 ± 0.089**
	**Sensitivity**	0.000 ± 0.000	0.000 ± 0.000	0.000 ± 0.000	0.000 ± 0.000	0.000 ± 0.000	0.000 ± 0.000	0.585 ± 0.097	0.420 ± 0.162	0.406 ± 0.159	0.512 ± 0.125
	**Specificity**	1.000 ± 0.000	1.000 ± 0.000	1.000 ± 0.000	1.000 ± 0.000	1.000 ± 0.000	1.000 ± 0.000	0.824 ± 0.059	0.984 ± 0.048	0.945 ± 0.050	0.915 ± 0.052
	**Accuracy**	0.905 ± 0.000	0.905 ± 0.000	0.905 ± 0.000	0.905 ± 0.000	0.905 ± 0.000	0.905 ± 0.000	0.714 ± 0.064	0.704 ± 0.074	0.696 ± 0.090	0.729 ± 0.072
MCRV (SBRT)	**AUC**	0.538 ± 0.099	0.538 ± 0.244	0.628 ± 0.287	0.695 ± 0.070	0.537 ± 0.122	0.749 ± 0.158	0.791 ± 0.033	**0.910 ± 0.034**	**0.888 ± 0.026**	**0.910 ± 0.042**
	**Sensitivity**	0.000 ± 0.000	0.000 ± 0.000	0.000 ± 0.000	0.000 ± 0.000	0.000 ± 0.000	0.000 ± 0.000	0.775 ± 0.059	0.714 ± 0.103	0.686 ± 0.051	0.619 ± 0.141
	**Specificity**	1.000 ± 0.000	1.000 ± 0.000	1.000 ± 0.000	0.982 ± 0.035	1.000 ± 0.000	1.000 ± 0.000	0.793 ± 0.042	0.898 ± 0.032	0.870 ± 0.051	0.962 ± 0.024
	**Accuracy**	0.905 ± 0.000	0.905 ± 0.000	0.905 ± 0.000	0.888 ± 0.032	0.905 ± 0.000	0.905 ± 0.000	0.784 ± 0.035	0.813 ± 0.048	0.785 ± 0.031	0.804 ± 0.066

Bold values represent the best statistically significant results.

**Table 4:** P-value of AUC results of the proposed MFC model compared to the PI model, R model, and DL model in LOOCV.

**Model 1**	**MFC (LR)**	**MFC (SVM)**	**MFC (RF)**	**MFC (LR)**	**MFC (SVM)**	**MFC (RF)**	**MFC (LR)**	**MFC (SVM)**	**MFC (RF)**
**Model 2**	**PI (LR)**	**PI (SVM)**	**PI (RF)**	**R (LR)**	**R (SVM)**	**R (RF)**	**DL**	**DL**	**DL**
**Surgery**	0.000*	0.000*	0.000*	0.000*	0.000*	0.012*	0.087	0.000*	0.000*
**SBRT**	0.000*	0.000*	0.000*	0.000*	0.000*	0.000*	0.000*	0.001*	0.000*

“*” marks the statistically significant difference.

For the LOOCV results, the proposed MFC model achieved the best prediction performance with the highest AUC for all three classifiers (for the surgery patient cohort/for the SBRT patient cohort): 0.858/0.868 for LR, 0.886/0.913 for SVM, and 0.895/0.907 for RF, respectively. These values were significantly higher than the other three comparison models. The PI models showed limited prediction performance with low values for all evaluation metrics. The R models showed improved AUC results (0.571/0.667 for LR, 0.559/0.639 for SVM, and 0.618/0.682 for RF, respectively), which demonstrated the significance of handcrafted radiomics analysis. However, the low sensitivity values (0.286-0.286/0.111-0.556) and high specificity values (0.671-0.711/0.813-0.907) suggest the R models cannot handle imbalanced datasets effectively: the classic radiomic analysis tends to predict all the test cases as non-local failures. Thus, the accuracy results (0.637-0.675/0.750-0.821) were from the biased models and cannot be accepted. The DL models showed a significant improvement in both sensitivity (0.428/0.556) and AUC results (0.809/0.843), but these results were still inferior to the proposed MFC model.

In the 100-fold MCRV, the proposed MFC model again showed the best prediction performance: the achieved AUC results (surgery/SBRT) of the MFC model were 0.742 ± 0.112/0.910 ± 0.034 for LR, 0.825 ± 0.061/0.888 ± 0.026 for SVM, and 0.792 ± 0.089/0.910 ± 0.042 for RF, respectively. The PI models again showed very low prediction results for three classifiers and two patient cohorts. Similarly, the R models showed limited AUC results for both patient cohorts in all three classifiers, while the RF approach achieved a slightly improved result than the LR and SVM classifiers. Due to the imbalanced outcome distribution in the dataset, the R models showed high specificity (1.000/0.982-1.000) with very low sensitivity (0.000-0.000/0.000-0.000). Although the DL model showed promising AUC results (0.702 ± 0.064/0.791 ± 0.033), these results were still significantly lower than the MFC model in the SVM and RF classifiers of the surgery cohort and in all three classifiers of the SBRT cohorts.

## 4 Discussion

In this study, we successfully developed an MFC model for early-stage NSCLC patients’ post-treatment local failure prediction using pre-treatment CT images. A key technical innovation is the integration of handcrafted radiomic image features, deep radiomic image features, and patient demographic information as a new patient-specific feature representation. Classic radiomic analysis extracts handcrafted image characteristics that reflect the underlying pathology and physiology within the GTV. The pre-trained deep-learning U-Net encoder was employed to extract generic anatomical representations from the tumor and its surrounding tissues. In addition to the pre-treatment CT-based image analysis, patient demographic information and treatment information were included in the prediction model as complementary clinical data. The combination of the features from multiple sources is expected to provide a more comprehensive description of tumor imaging characteristics and overall patient physiology, which may aid in the local failure prediction in early-stage NSCLC.

The clinical outcomes prediction of patients with early-stage NSCLC has been widely reported with various techniques. Many studies have reported that patient demographic information (e.g., TNM staging system) can be utilized for clinical outcome prediction (21, 22). In this work, only gender, age, tumor volume, and CCI (plus prescription dose and the fractionation scheme for the SBRT cohort) were investigated due to the data availability. However, the compromised AUC and accuracy values of the PI models (for both patient cohorts) suggest that the collected demographic information and treatment information only carries limited predictive power for local failure. More patient information and treatment information are needed to investigate the full potential of patient demographic information and clinical information. In contrast, the classic radiomics analysis is a widely adopted image analysis method. The performance of R models confirms the prognostic significance of radiomics in local failure prediction of early-stage NSCLC, and the achieved AUC results (highest AUC results are 0.652 ± 0.183 and 0.749 ± 0.158 for surgery and SBRT cohort in 100-fold MCRV, respectively) are in line with previous studies (25, 27–31). However, studies have reported that classic radiomic features are sensitive to image acquisition parameters (e.g., CT scanner, reconstruction algorithms, resolution, slice thickness, etc.) and pre-processing workflows (e.g., discretization, interpolation, etc.) (54, 55). The effective management of imbalanced data can also be challenging for classic radiomic analysis. The low robustness (e.g., large red shaded area in **Figure 5**) and low sensitivity (e.g., surgery: 0.000-0.000 and SBRT: 0.000-0.000 in 100-fold MCRV) in the R models’ results confirm these limitations. As an advanced computational approach, deep learning has been recently applied to clinical outcome prediction in early-stage NSCLC. In this study, the pre-trained Genesis model extracted the generic anatomical representation as a transfer learning technique without requiring a large amount of data. The adoption of data augmentation further improved data utilization. Consequently, the DL models showed improved sensitivity (e.g., surgery: 0.585 ± 0.097 and SBRT: 0.775 ± 0.059 for 100-fold MCRV) and AUC results (e.g., surgery: 0.702 ± 0.064 and SBRT: 0.791 ± 0.033 for 100-fold MCRV) compared to the PI and R models. Nevertheless, the deep radiomic features are highly data-dependent and are directly learned by the neural networks without human supervision; these make the extracted features incompatible with human experts’ interpretation within the existing knowledge domain (56). In the proposed MFC model, the extracted deep radiomic features were utilized in the supervised machine learning classifiers, along with handcrafted radiomic features and patient demographic information. The obtained prediction performance is significantly higher than PI/R/DL comparison models.

Two validation methods, LOOCV and 100-fold MCRV, were employed in this study. Compared to the 100-fold MCRV, the LOOCV showed higher AUC values for the MFC model across three classifiers and two patient cohorts. In LOOCV, the training-test split is not randomized, and each sample has the potential to represent the entire test set, resulting in less bias in performance measurements. Therefore, LOOCV has been adopted in studies with small sample sizes (57). In addition, compared to the 100-fold MCRV following 70%-30% training test assignments, LOOCV produced up to 40%/50% (surgery/SBRT) more local failure samples for training in this study. The increased sample size of local failures provided a larger and more balanced training set, which is essential for improving sensitivity and overall prediction performance. In 100-fold MCRV, the models were trained and evaluated with 100 versions of random validation sample assignments. The variation of the prediction performance (shown as the shaded area in **Figure 4**) can be considered the quantification for model uncertainty. Uncertainty provides additional information relating to the model’s robustness for a single ROC curve. The adoption of both validation methods allows a more objective evaluation of the model performance differences (58).

The COVID-19 pandemic has presented unprecedented challenges to cancer treatment. Surgery remains the current standard of care modality for early-stage NSCLC, but it is frequently delayed or even canceled due to the pandemic (59). The average delay in surgery due to COVID-19 has been reported to be four weeks (60). As a safe, effective, and efficient treatment technique, SBRT holds the potential to be considered the standard of care for early-stage NSCLC treatment in the absence of surgery capacity (61, 62). This is currently being prospectively evaluated in the VALOR study (63), a randomized clinical trial comparing surgery to SBRT in operable patients. In this study, we demonstrated the local failure prediction models for both surgery and SBRT patient cohorts. An interesting finding is that the MFC model built in the SBRT patient cohort achieved a higher AUC compared to the surgical cohort, for both LOOCV and 100-fold MCRV. However, the current results from MFC may be insufficient to evaluate the treatment modality effectiveness due to the relatively small data sample size and not-curated baseline patient characteristics. A unified treatment risk modeling across both patient cohorts is challenging: first, the definition of local failure in surgical and SBRT treatments can be a potential factor contributing to differences in predictive performance. The local failure in SBRT refers to local recurrence at the initial treatment site. Previous studies have demonstrated that dense and homogeneous image content within GTV regions is associated with larger and denser tumors, resulting in a poorer prognosis (64, 65). In our MFC model, both classic radiomic and deep learning analysis focused on image quantification near the center of the tumor, which may better capture the image features associated with SBRT treatment outcomes. In addition, the pre-treatment CT images in the SBRT patient cohort demonstrated a more uniform in-plane resolution and slice thickness than the surgery cohort (see **Table 1**), which could also lead to differences in prediction performance.

We note that two retrospective patient cohorts included in this study were relatively small and were not collected prospectively to be comparable. Therefore, the developed MFC models are considered completely independent in two patient cohorts, and the application of the MFC model to local failure prediction is only a proof of concept. The performance of the prediction model can be significantly limited by the data availability. The substantial well-annotated early-stage NSCLC datasets with balanced outcome distributions are rare in real-world medical imaging. Therefore, our current work focused more on the technical development side. The initial results suggest that, when compared with state-of-the-art modeling, combining feature information from multiple sources could potentially improve the prediction ability of local failure in early-stage NSCLC patients. In a future large dataset with balanced outcome distribution and well-curated clinical data collection, the developed MFC holds the potential to identify patient-specific local recurrence risk information for both treatment modalities simultaneously; this may allow individual-based treatment strategy modification for better outcomes. In addition, the MFC model in this work fused multiple sources of information by direct concatenation. However, the image features have significantly higher dimensions than demographics, which may not allow the prediction model to fully leverage the contribution of each information source (40). The equal-dimension comparison may better characterize the role of patient demographic information, treatment information, handcrafted radiomic feature, deep radiomic feature, and their combinations (66). The explainable AI model is another future research direction to identify the contribution of each input feature to the final prediction and acquire optimal fusion results. Such feature contribution analysis may aid in understanding the internal decision-making process of local failure prediction models, and ultimately provide additional information for the potential clinical applications of the developed MFC model.

## 5 Conclusion

We successfully developed an MFC model that combines feature information from multiple sources for proof-of-concept prediction of local failure in patients with surgical and SBRT early-stage NSCLC. Initial results suggested that incorporating pre-treatment patient information from multiple sources improves the ability to predict the risk of local failure. Future works in large patient cohorts are desired to further evaluate the performance of MFC model.

## Data availability statement

The raw data supporting the conclusions of this article will be made available by the authors upon reasonable request, subject to de-identification procedures to ensure compliance with ethical guidelines and regulations for protecting patient privacy. 

## Author contributions

ZY developed the model and performed the computations. ZY and CW wrote the manuscript with support from YW, KL, HZ, BA, CK, BT. F-FY supervised the project. All authors contributed to the article and approved the submitted version.

## References

[1] Sung H Ferlay J Siegel RL Laversanne M Soerjomataram I Jemal A. Global cancer statistics 2020: GLOBOCAN estimates of incidence and mortality worldwide for 36 cancers in 185 countries. CA: Cancer J Clin (2021) 71(3):209–49. doi: 10.3322/caac.21660 33538338

[2] Ferlay J Colombet M Soerjomataram I Parkin DM Piñeros M Znaor A. Cancer statistics for the year 2020: An overview. Int J Cancer (2021) 149(4):778–89. doi: 10.1002/ijc.33588 33818764

[3] Molina JR Yang P Cassivi SD Schild SE Adjei AA. Non-small cell lung cancer: epidemiology, risk factors, treatment, and survivorship. Mayo clinic Proc (2008) 83(5):584–94. doi: 10.4065/83.5.584 PMC2718421 18452692

[4] Abbosh C Birkbak NJ Swanton C. Early stage NSCLC—challenges to implementing ctDNA-based screening and MRD detection. Nat Rev Clin Oncol (2018) 15(9):577–86. doi: 10.1038/s41571-018-0058-3 29968853

[5] Rosell R Karachaliou N. Optimizing lung cancer treatment approaches. Nat Rev Clin Oncol (2015) 12(2):75–6. doi: 10.1038/nrclinonc.2014.225 25533943

[6] Zappa C Mousa SA. Non-small cell lung cancer: current treatment and future advances. Trans Lung Cancer Res (2016) 5(3):288. doi: 10.21037/tlcr.2016.06.07 PMC4931124 27413711

[7] Postmus P Kerr K Oudkerk M Senan S Waller D Vansteenkiste J. Early and locally advanced non-small-cell lung cancer (NSCLC): ESMO Clinical Practice Guidelines for diagnosis, treatment and follow-up. Ann Oncol (2017) 28:iv1–iv21. doi: 10.1093/annonc/mdx222 28881918

[8] Kastelijn EA El Sharouni SY Hofman FN Van Putte BP Monninkhof EM Van Vulpen M. Clinical outcomes in early-stage NSCLC treated with stereotactic body radiotherapy versus surgical resection. Anticancer Res (2015) 35(10):5607–14. 26408733

[9] Raz DJ Zell JA Ou SI Gandara DR Anton-Culver H Jablons DM. Natural history of stage I non-small cell lung cancer: implications for early detection. Chest (2007) 132(1):193–9. doi: 10.1378/chest.06-3096 17505036

[10] Wisnivesky JP Bonomi M Henschke C Iannuzzi M McGinn T. Radiation therapy for the treatment of unresected stage I-II non-small cell lung cancer. Chest (2005) 128(3):1461–7. doi: 10.1378/chest.128.3.1461 16162744

[11] Timmerman RD Paulus R Pass HI Gore E Edelman MJ Galvin JM. RTOG 0618: Stereotactic body radiation therapy (SBRT) to treat operable early-stage lung cancer patients. Am Soc Clin Oncol (2013). doi: 10.1200/jco.2013.31.15_suppl.7523 PMC6117102 29852037

[12] Sebastian NT Xu-Welliver M Williams TM. Stereotactic body radiation therapy (SBRT) for early stage non-small cell lung cancer (NSCLC): contemporary insights and advances. J Thorac Dis (2018) 10(Suppl 21):S2451. doi: 10.21037/jtd.2018.04.52 30206491 PMC6123192

[13] Tandberg DJ Tong BC Ackerson BG Kelsey CR. Surgery versus stereotactic body radiation therapy for stage I non–small cell lung cancer: a comprehensive review. Cancer (2018) 124(4):667–78. doi: 10.1002/cncr.31196 29266226

[14] Ball D Mai GT Vinod S Babington S Ruben J Kron T. Stereotactic ablative radiotherapy versus standard radiotherapy in stage 1 non-small-cell lung cancer (TROG 09.02 CHISEL): a phase 3, open-label, randomised controlled trial. Lancet Oncol (2019) 20(4):494–503. doi: 10.1016/S1470-2045(18)30896-9 30770291

[15] Schneider BJ Daly ME Kennedy EB Antonoff MB Broderick S Feldman J. Stereotactic body radiotherapy for early-stage non–small-cell lung cancer: American society of clinical oncology endorsement of the American society for radiation oncology evidence-based guideline. J Clin Oncol (2018) 36(7):710–9. doi: 10.1200/JCO.2017.74.9671 29106810

[16] Zheng X Schipper M Kidwell K Lin J Reddy R Ren Y. Survival outcome after stereotactic body radiation therapy and surgery for stage I non-small cell lung cancer: a meta-analysis. Int J Radiat Oncol Biol Phys (2014) 90(3):603–11. doi: 10.1016/j.ijrobp.2014.05.055 25052562

[17] Spencer KL Kennedy MP Lummis KL Ellames DA Snee M Brunelli A. Surgery or radiotherapy for stage I lung cancer? An intention-to-treat analysis. Eur Respir J (2019) 53(6). doi: 10.1183/13993003.01568-2018 30635294

[18] Chen H Laba JM Boldt RG Goodman CD Palma DA Senan S. Stereotactic ablative radiation therapy versus surgery in early lung cancer: a meta-analysis of propensity score studies. Int J Radiat Oncol Biol Phys (2018) 101(1):186–94. doi: 10.1016/j.ijrobp.2018.01.064 29619964

[19] Vachani A Sequist LV Spira A. AJRCCM: 100-year anniversary. The shifting landscape for lung cancer: past, present, and future. Am J Respir Crit Care Med (2017) 195(9):1150–60. doi: 10.1164/rccm.201702-0433CI PMC5439022 28459327

[20] Jiao Z Li H Xiao Y Dorsey J Simone CB Feigenberg S. Integration of deep learning radiomics and counts of circulating tumor cells improves prediction of outcomes of early stage NSCLC patients treated with stereotactic body radiation therapy. Int J Radiat Oncol Biol Phys (2022) 112(4):1045–54. doi: 10.1016/j.ijrobp.2021.11.006 PMC9074888 34775000

[21] Asamura H Chansky K Crowley J Goldstraw P Rusch VW Vansteenkiste JF. The International Association for the Study of Lung Cancer Lung Cancer Staging Project: proposals for the revision of the N descriptors in the forthcoming 8th edition of the TNM classification for lung cancer. J Thorac Oncol (2015) 10(12):1675–84. doi: 10.1097/JTO.0000000000000678 26709477

[22] Chaft JE Rimner A Weder W Azzoli CG Kris MG Cascone T. Evolution of systemic therapy for stages I–III non-metastatic non-small-cell lung cancer. Nat Rev Clin Oncol (2021) 18(9):547–57. doi: 10.1038/s41571-021-00501-4 PMC9447511 33911215

[23] Aerts HJWL Velazquez ER Leijenaar RTH Parmar C Grossmann P Carvalho S. Decoding tumour phenotype by noninvasive imaging using a quantitative radiomics approach. Nat Commun (2014) 5(1). doi: 10.1038/ncomms5644 PMC4059926 24892406

[24] Lambin P Rios-Velazquez E Leijenaar R Carvalho S Van Stiphout RG Granton P. Radiomics: extracting more information from medical images using advanced feature analysis. Eur J Cancer (2012) 48(4):441–6. doi: 10.1016/j.ejca.2011.11.036 PMC4533986 22257792

[25] Li H Galperin-Aizenberg M Pryma D Simone CB II Fan Y. Unsupervised machine learning of radiomic features for predicting treatment response and overall survival of early stage non-small cell lung cancer patients treated with stereotactic body radiation therapy. Radiotherapy Oncol (2018) 129(2):218–26. doi: 10.1016/j.radonc.2018.06.025 PMC6261331 30473058

[26] Lafata KJ Wang Y Konkel B Yin F-F Bashir MR. Radiomics: a primer on high-throughput image phenotyping. Abdominal Radiol (2021) p:1–17. doi: 10.1007/s00261-021-03254-x 34435228

[27] Starkov P Aguilera TA Golden DI Shultz DB Trakul N Maxim PG. The use of texture-based radiomics CT analysis to predict outcomes in early-stage non-small cell lung cancer treated with stereotactic ablative radiotherapy. Br J Radiol (2019) 92(1094):20180228. doi: 10.1259/bjr.20180228 30457885 PMC6404825

[28] Huang Y Liu Z He L Chen X Pan D Ma Z. Radiomics signature: a potential biomarker for the prediction of disease-free survival in early-stage (I or II) non-small cell lung cancer. Radiology (2016) 281(3):947–957. doi: 10.1148/radiol.2016152234 27347764

[29] Emaminejad N Yan S Wang Y Qian W Guan Y Zheng B. Applying a radiomics approach to predict prognosis of lung cancer patients. In: Medical imaging 2016: computer-aided diagnosis. SPIE (2016) 9785:352–358.

[30] Huynh E Coroller TP Narayan V Agrawal V Hou Y Romano J. CT-based radiomic analysis of stereotactic body radiation therapy patients with lung cancer. Radiotherapy Oncol (2016) 120(2):258–66. doi: 10.1016/j.radonc.2016.05.024 27296412

[31] Wang T She Y Yang Y Liu X Chen S Zhong Y. Radiomics for survival risk stratification of clinical and pathologic stage IA pure-solid non–small cell lung cancer. Radiology (2022) 302(2):425–34. doi: 10.1148/radiol.2021210109 34726531

[32] Xu Y Hosny A Zeleznik R Parmar C Coroller T Franco I. Deep learning predicts lung cancer treatment response from serial medical imagingLongitudinal deep learning to track treatment response. Clin Cancer Res (2019) 25(11):3266–75. doi: 10.1158/1078-0432.CCR-18-2495 PMC6548658 31010833

[33] Hu Z Yang Z Lafata KJ Yin FF Wang C. A radiomics-boosted deep-learning model for COVID-19 and non-COVID-19 pneumonia classification using chest x-ray images. Med Phys (2022) 49(5):3213–22. doi: 10.1002/mp.15582 PMC9088469 35263458

[34] Yang Z Lafata K Vaios E Hu Z Mullikin T Yin F-F. Quantifying U-net uncertainty in multi-parametric MRI-based glioma segmentation by spherical image projection. arXiv (2022) 2210.06512. doi: 10.48550/arXiv.2210.06512 PMC10925552 37696029

[35] Yang Z Lafata KJ Chen X Bowsher J Chang Y Wang C. Quantification of lung function on CT images based on pulmonary radiomic filtering. Med Phys (2022) 49(11):7278–86. doi: 10.1002/mp.15837 35770964

[36] Xu Z Li S Xu J Liu J Luo X Zhang Y. LDFR: Learning deep feature representation for software defect prediction. J Syst Software (2019) 158:110402. doi: 10.1016/j.jss.2019.110402

[37] Zhong Y She Y Deng J Chen S Wang T Yang M. Deep learning for prediction of N2 metastasis and survival for clinical stage I non–small cell lung cancer. Radiology (2022) 302(1):200–11. doi: 10.1148/radiol.2021210902 34698568

[38] Lian J Deng J Hui ES Koohi-Moghadam M She Y Chen C. Early stage NSCLS patients’ prognostic prediction with multi-information using transformer and graph neural network model. Elife (2022) 11:e80547. doi: 10.7554/eLife.80547 36194194 PMC9531948

[39] Ackerson BG Tong BC Hong JC Gu L Chino J Trotter JW. Stereotactic body radiation therapy versus sublobar resection for stage I NSCLC. Lung Cancer (2018) 125:185–91. doi: 10.1016/j.lungcan.2018.09.020 30429018

[40] Wang Y Li X Konanur M Konkel B Seyferth E Brajer N. Towards optimal deep fusion of imaging and clinical data via a model-based description of fusion quality. Med Phys (2022). doi: 10.1002/mp.16181 36548913

[41] Birim Ö Kappetein AP Bogers AJ. Charlson comorbidity index as a predictor of long-term outcome after surgery for nonsmall cell lung cancer. Eur J cardio-thoracic Surg (2005) 28(5):759–62. doi: 10.1016/j.ejcts.2005.06.046 16157485

[42] Fedor D Johnson WR Singhal S. Local recurrence following lung cancer surgery: incidence, risk factors, and outcomes. Surg Oncol (2013) 22(3):156–61. doi: 10.1016/j.suronc.2013.04.002 PMC4131285 23702313

[43] Kumar SS McGarry RC. Management of local recurrences and regional failure in early stage non-small cell lung cancer after stereotactic body radiation therapy. Trans Lung Cancer Res (2019) 8(Suppl 2):S213. doi: 10.21037/tlcr.2019.09.06 PMC6795579 31673526

[44] Duron L Balvay D Perre Vande S Bouchouicha A Savatovsky J Sadik J-C. Gray-level discretization impacts reproducible MRI radiomics texture features. PloS One (2019) 14(3):e0213459. doi: 10.1371/journal.pone.0213459 30845221 PMC6405136

[45] Crombé A Kind M Fadli D Le Loarer F Italiano A Buy X. Intensity harmonization techniques influence radiomics features and radiomics-based predictions in sarcoma patients. Sci Rep (2020) 10(1):1–13. doi: 10.1038/s41598-020-72535-0 32968131 PMC7511974

[46] Zwanenburg A Vallières M Abdalah MA Aerts HJWL Andrearczyk V Apte A. The image biomarker standardization initiative: standardized quantitative radiomics for high-throughput image-based phenotyping. Radiology (2020) 295(2):328–38. doi: 10.1148/radiol.2020191145 PMC7193906 32154773

[47] Chang Y Lafata K Wang C Duan X Geng R Yang Z. Digital phantoms for characterizing inconsistencies among radiomics extraction toolboxes. Biomed Phys Eng Express (2020) 6(2):025016. doi: 10.1088/2057-1976/ab779c 33438642

[48] Ronneberger O Fischer P Brox T. U-net: Convolutional networks for biomedical image segmentation. In Medical Image Computing and Computer-Assisted Intervention–MICCAI 2015: 18th International Conference, Munich, Germany, October 5-9, 2015, Proceedings, Part III. Springer International Publishing (2015) 18:234–241. doi: 10.1007/978-3-319-24574-4_28

[49] Zhou Z Sodha V Pang J Gotway MB Liang J. Models genesis. Med image Anal (2021) 67:101840. doi: 10.1016/j.media.2020.101840 33188996 PMC7726094

[50] Balagurunathan Y Kumar V Gu Y Kim J Wang H Liu Y. Test–retest reproducibility analysis of lung CT image features. J digital Imaging (2014) 27(6):805–23. doi: 10.1007/s10278-014-9716-x PMC4391075 24990346

[51] Schabath MB Massion PP Thompson ZJ Eschrich SA Balagurunathan Y Goldof D. Differences in patient outcomes of prevalence, interval, and screen-detected lung cancers in the CT arm of the National Lung Screening Trial. PloS One (2016) 11(8):e0159880. doi: 10.1371/journal.pone.0159880 27509046 PMC4980050

[52] Wong T-T. Performance evaluation of classification algorithms by k-fold and leave-one-out cross validation. Pattern Recognition (2015) 48(9):2839–46. doi: 10.1016/j.patcog.2015.03.009

[53] Xu Q-S Liang Y-Z. Monte Carlo cross validation. Chemometrics Intelligent Lab Syst (2001) 56(1):1–11. doi: 10.1016/S0169-7439(00)00122-2

[54] Roy S Whitehead TD Quirk JD Salter A Ademuyiwa FO Li S. Optimal co-clinical radiomics: Sensitivity of radiomic features to tumour volume, image noise and resolution in co-clinical T1-weighted and T2-weighted magnetic resonance imaging. EBioMedicine (2020) 59:102963. doi: 10.1016/j.ebiom.2020.102963 32891051 PMC7479492

[55] Shi L He Y Yuan Z Benedict S Valicenti R Qiu J. Radiomics for response and outcome assessment for non-small cell lung cancer. Technol Cancer Res Treat (2018) 17:1533033818782788. doi: 10.1177/1533033818782788 29940810 PMC6048673

[56] Dash T Chitlangia S Ahuja A Srinivasan A. A review of some techniques for inclusion of domain-knowledge into deep neural networks. Sci Rep (2022) 12(1):1–15. doi: 10.1038/s41598-021-04590-0 35058487 PMC8776800

[57] Airola A Pahikkala T Waegeman W Baets De B Salakoski T. A comparison of AUC estimators in small-sample studies. Mach Learn Syst Biol (2009), pp 3–13.

[58] Hüllermeier E Waegeman W. Aleatoric and epistemic uncertainty in machine learning: An introduction to concepts and methods. Mach Learn (2021) 110(3):457–506. doi: 10.1007/s10994-021-05946-3

[59] Burki TK. Cancer guidelines during the COVID-19 pandemic. Lancet Oncol (2020) 21(5):629–30. doi: 10.1016/S1470-2045(20)30217-5 PMC7270910 32247319

[60] Kovoor JG Scott NA Tivey DR Babidge WJ Scott DA Beavis VS. Proposed delay for safe surgery after COVID-19. ANZ J Surg (2021) 91(4):495–506. doi: 10.1111/ans.16682 33656269 PMC8014540

[61] Passaro A Addeo A Von Garnier C Blackhall F Planchard D Felip E. ESMO Management and treatment adapted recommendations in the COVID-19 era: Lung cancer. ESMO Open (2020) 5(Suppl 3). doi: 10.1136/esmoopen-2020-000820 PMC7319703 32581069

[62] Chang JY Li Q-Q Xu Q-Y Allen PK Rebueno N Gomez DR. Stereotactic ablative radiation therapy for centrally located early stage or isolated parenchymal recurrences of non-small cell lung cancer: how to fly in a “no fly zone”. Int J Radiat Oncol Biol Phys (2014) 88(5):1120–8. doi: 10.1016/j.ijrobp.2014.01.022 24661665

[63] MoGhanaki D Hagan M. Strategic initiatives for veterans with lung cancer. Federal Practitioner (2020) 37(Suppl 4):S76. doi: 10.12788/fp.0019 32908355 PMC7473723

[64] Lafata KJ Hong JC Geng R Ackerson BG Liu J-G Zhou Z. Association of pre-treatment radiomic features with lung cancer recurrence following stereotactic body radiation therapy. Phys Med Biol (2019) 64(2):025007. doi: 10.1088/1361-6560/aaf5a5 30524018

[65] Ye J Chang J Li Z Wernicke A Nori D Parashar B. Tumor density, size, and histology in the outcome of stereotactic body radiation therapy for early-stage non-small-cell lung cancer: A single-institution experience. Ann Meeting Am Radium Soc (2015). 25930849

[66] Yang Z Hu Z Ji H Lafata K Vaios E Floyd S. A neural ordinary differential equation model for visualizing deep neural network behaviors in multi-parametric MRI-based glioma segmentation. Med Phys (2023) 50:4825–4838. doi: 10.1002/mp.16286 PMC10440249 36840621

