# Supplementary material for: Development of a multi-feature-combined model: proof-of-concept with application to local failure prediction of post-SBRT or surgery early-stage NSCLC patients
Source: Front Oncol. 2023 Sep 13;13:1185771. doi: 10.3389/fonc.2023.1185771 (PMC10534017; doi:10.3389/fonc.2023.1185771)
Supplement: Supplementary file 1 [file Table_1.docx]

Supplementary Materials for the “A Multi-Feature-Combined Model for Post SBRT or Surgery Local Failure Prediction using Pre-treatment CT in Early-Stage NSCLC Patients.”

**Table I: 105 radiomics features included in this study.**

| **3D shape-based morphological features** | | **GLCOM-Based Features** | |
| --- | --- | --- | --- |
| 1 | Volume | 1 | Auto Correlation |
| 2 | **Surface Area** | 2 | Cluster Prominence |
| 3 | **Surface Area to Volume ratio** | 3 | Cluster Shade |
| 4 | **Sphericity** | 4 | Cluster Tendency |
| 5 | **Compactness 1** | 5 | Contrast |
| 6 | **Compactness 2** | 6 | Correlation |
| 7 | **Spherical Disproportion** | 7 | Differential Entropy |
| 8 | **Maximum 3D diameter** | 8 | Dissimilarity |
| 9 | **Major Axis Length** | 9 | Joint Energy / Angular Second Moment |
| 10 | **Minor Axis Length** | 10 | Joint Entropy |
| 11 | **Least Axis Length** | 11 | Homogeneity 1 / Inverse Difference |
| 12 | **Elongation** | 12 | Homogeneity 2 / Inverse Difference Moment |
| 13 | **Flatness** | 13 | Info Measure Correlation 1 |
| **Intensity-Based Features** | | 14 | Info Measure Correlation 2 |
|  |  | 15 | Inverse Difference Moment Normalized |
| 1 | Mean | 16 | Inverse Difference Normalized |
| 2 | Variance | 17 | Inverse Variance |
| 3 | Skewness | 18 | Joint Maximum |
| 4 | Intensity Histogram Kurtosis | 19 | Sum Average |
| 5 | Median | 20 | Sum Entropy |
| 6 | Minimum Grey Level | 21 | Sum Variance |
| 7 | 10th Percentile | 22 | Joint Variance |
| 8 | 90th Percentile | **GLRLM-Based Features** | |
| 9 | Maximum Grey Level |  |  |
| 10 | Interquartile Range | 1 | Short Run Emphasis |
| 11 | Range | 2 | Long Run Emphasis |
| 12 | Mean Absolute Deviation | 3 | Gray Level Non-Uniformity |
| 13 | Robust Mean Absolute Deviation | 4 | Gray Level Non-Uniformity Normalized |
| 14 | Median Absolute Deviation | 5 | Run Length Non-Uniformity |
| 15 | Coefficient of Variation | 6 | Run Length Non-Uniformity Normalized |
| 16 | Quartile Coefficient of Dispersion | 7 | Run Percentage |
| 17 | Energy | 8 | Low Gray Level Run Emphasis |
| 18 | Root Mean Square | 9 | High Gray Level Run Emphasis |
| **Histogram-Based Features** | | 10 | Short Run Low Gray Level Emphasis |
|  |  | 11 | Short Run High Gray Level Emphasis |
| 1 | Intensity Histogram Mean | 12 | Long Run Low Gray Level Emphasis |
| 2 | Intensity Histogram Variance | 13 | Long Run High Gray Level Emphasis |
| 3 | Intensity Histogram Skewness | 14 | Grey Level Variance |
| 4 | Intensity Histogram Kurtosis | 15 | Run Length Variance |
| 5 | Intensity Histogram Median | 16 | Run Entropy |
| 6 | Intensity Histogram Minimum Grey Level | **GLSZM-Based Features** | |
| 7 | Intensity Histogram 10th Percentile |  |  |
| 8 | Intensity Histogram 90th Percentile | 1 | Small Zone Emphasis |
| 9 | Intensity Histogram Maximum Grey Level | 2 | Large Zone Emphasis |
| 10 | Intensity Histogram Interquartile Range | 3 | Gray Level Non-Uniformity |
| 11 | Intensity Histogram Range | 4 | Gray Level Non-Uniformity Normalized |
| 12 | Intensity Histogram Mean Absolute Deviation | 5 | Size Zone Non-Uniformity |
| 13 | Intensity Histogram Robust Mean Absolute Deviation | 6 | Size Zone Non-Uniformity Normalized |
| 14 | Intensity Histogram Median Absolute Deviation | 7 | Zone Percentage |
| 15 | Intensity Histogram Coefficient of Variation | 8 | Low Gray Level Size Emphasis |
| 16 | Intensity Histogram Quartile Coefficient of Dispersion | 9 | High Gray Level Size Emphasis |
| 17 | Intensity Histogram Entropy | 10 | Small Size Low Gray Level Emphasis |
| 18 | Intensity Histogram Uniformity | 11 | Small Size High Gray Level Emphasis |
| 19 | Maximum Histogram Gradient | 12 | Large Size Low Gray Level Emphasis |
| 20 | Minimum Histogram Gradient | 13 | Large Size High Gray Level Emphasis |
|  |  | 14 | Gray Level Variance |
|  |  | 15 | Zone Size Variance |
|  |  | 16 | Zone Size Entropy |

**Table II: Surgery Patient Data.**

| Subject | Local Failure | Date of Procedure | Age | Sex | CCI | Tumor Size (cc) | Smoke HX | CT FOV (mm) | CT Pixel Spacing (mm) | CT Manufacturer | CT Model |
| --- | --- | --- | --- | --- | --- | --- | --- | --- | --- | --- | --- |
| Subject #1 | No | January 2011 | 74 | Female | 4 | 1.8 | 2 | 0.98, 0.98, 3.75 | 500, 500, 1121 | Discovery 690 | GE MEDICAL SYSTEMS |
| Subject #2 | No | January 2013 | 69 | Male | 1 | 1.4 | 2 | 0.98, 0.98, 3.75 | 500, 500, 1068 | Discovery STE | GE MEDICAL SYSTEMS |
| Subject #3 | No | August 2012 | 67 | Female | 4 | 1.3 | 1 | 0.98, 0.98, 3.75 | 500, 500, 1500 | Discovery STE | GE MEDICAL SYSTEMS |
| Subject #4 | Yes | April 2011 | 88 | Male | 2 | 1.2 | 2 | 0.74, 0.74, 1.25 | 381, 381, 241 | Discovery CT750 HD | GE MEDICAL SYSTEMS |
| Subject #5 | No | January 2011 | 74 | Female | 2 | 4.3 | 2 | 0.98, 0.98, 3.75 | 500, 500, 1121 | Discovery 690 | GE MEDICAL SYSTEMS |
| Subject #6 | No | October 2012 | 59 | Male | 3 | 0.7 | 2 | 0.80, 0.80, 5.00 | 410, 410, 360 | Discovery CT750 HD | GE MEDICAL SYSTEMS |
| Subject #7 | No | July 2009 | 77 | Male | 3 | 0.9 | 2 | 0.98, 0.98, 3.75 | 500, 500, 1121 | Discovery 690 | GE MEDICAL SYSTEMS |
| Subject #8 | No | February 2013 | 83 | Female | 1 | 1 | 0 | 0.68, 0.68, 0.63 | 347, 347, 250 | LightSpeed VCT | GE MEDICAL SYSTEMS |
| Subject #9 | No | June 2013 | 74 | Female | 1 | 2 | 2 | 0.70, 0.70, 0.63 | 360, 360, 315 | Discovery CT750 HD | GE MEDICAL SYSTEMS |
| Subject #10 | No | September 2012 | 54 | Female | 3 | 2.4 | 0 | 0.98, 0.98, 3.75 | 500, 500, 941 | Discovery STE | GE MEDICAL SYSTEMS |
| Subject #11 | No | January 2013 | 69 | Female | 0 | 1 | 0 | 0.98, 0.98, 3.75 | 500, 500, 986 | Discovery 690 | GE MEDICAL SYSTEMS |
| Subject #12 | No | November 2010 | 66 | Male | 2 | 2 | 0 | 0.98, 0.98, 3.75 | 500, 500, 1121 | Discovery 690 | GE MEDICAL SYSTEMS |
| Subject #13 | No | January 2010 | 68 | Male | 3 | 2.8 | 2 | 0.70, 0.70, 5.00 | 356, 356, 295 | SOMATOM Definition Flash | SIEMENS |
| Subject #14 | No | March 2013 | 68 | Male | 2 | 1.1 | 1 | 0.98, 0.98, 3.75 | 500, 500, 941 | Discovery STE | GE MEDICAL SYSTEMS |
| Subject #15 | No | February 2011 | 80 | Female | 3 | 1.1 | 2 | 0.98, 0.98, 3.75 | 500, 500, 1068 | Discovery STE | GE MEDICAL SYSTEMS |
| Subject #16 | No | January 2011 | 50 | Female | 6 | 1.3 | 2 | 0.55, 0.55, 5.00 | 280, 280, 295 | LightSpeed VCT | GE MEDICAL SYSTEMS |
| Subject #17 | No | May 2011 | 55 | Female | 2 | 0.8 | 0 | 0.61, 0.61, 5.00 | 310, 310, 320 | LightSpeed16 | GE MEDICAL SYSTEMS |
| Subject #18 | No | May 2010 | 68 | Female | 3 | 1.4 | 0 | 0.63, 0.63, 5.00 | 320, 320, 340 | LightSpeed16 | GE MEDICAL SYSTEMS |
| Subject #19 | No | October 2010 | 66 | Male | 2 | 1.8 | 1 | 0.98, 0.98, 3.75 | 500, 500, 1121 | Discovery 690 | GE MEDICAL SYSTEMS |
| Subject #20 | No | January 2012 | 63 | Male | 3 | 1.5 | 2 | 0.98, 0.98, 3.75 | 500, 500, 1121 | Discovery 690 | GE MEDICAL SYSTEMS |
| Subject #21 | No | August 2013 | 73 | Female | 1 | 1 | 1 | 0.68, 0.68, 0.60 | 348, 348, 313 | SOMATOM Definition Flash | SIEMENS |
| Subject #22 | No | June 2014 | 63 | Female | 0 | 1.3 | 1 | 0.98, 0.98, 3.75 | 500, 500, 813 | Discovery STE | GE MEDICAL SYSTEMS |
| Subject #23 | Yes | August 2014 | 67 | Female | 1 | 1.5 | 2 | 0.61, 0.61, 0.63 | 310, 310, 330 | Discovery CT750 HD | GE MEDICAL SYSTEMS |
| Subject #24 | No | September 2014 | 65 | Male | 1 | 1.7 | 1 | 0.74, 0.74, 0.60 | 379, 379, 330 | SOMATOM Definition Flash | SIEMENS |
| Subject #25 | No | October 2014 | 57 | Male | 3 | 1.4 | 2 | 0.98, 0.98, 3.75 | 500, 500, 1068 | Discovery STE | GE MEDICAL SYSTEMS |
| Subject #26 | No | October 2014 | 76 | Male | 4 | 3.8 | 2 | 0.98, 0.98, 3.75 | 500, 500, 1121 | Discovery 690 | GE MEDICAL SYSTEMS |
| Subject #27 | No | July 2013 | 61 | Male | 2 | 1.1 | 1 | 1.37, 1.37, 3.75 | 700, 700, 1256 | Discovery 690 | GE MEDICAL SYSTEMS |
| Subject #28 | No | May 2011 | 63 | Female | 2 | 1.6 | 2 | 0.98, 0.98, 3.75 | 500, 500, 1121 | Discovery 690 | GE MEDICAL SYSTEMS |
| Subject #29 | Yes | July 2014 | 50 | Female | 2 | 1.4 | 2 | 0.64, 0.64, 5.00 | 330, 330, 330 | LightSpeed Xtra | GE MEDICAL SYSTEMS |
| Subject #30 | No | April 2013 | 70 | Male | 4 | 3.2 | 1 | 0.98, 0.98, 3.75 | 500, 500, 1121 | Discovery 690 | GE MEDICAL SYSTEMS |
| Subject #31 | No | February 2013 | 78 | Female | 1 | 3.5 | 2 | 0.98, 0.98, 3.75 | 500, 500, 986 | Discovery 690 | GE MEDICAL SYSTEMS |
| Subject #32 | No | December 2010 | 73 | Female | 3 | 1 | 1 | 0.98, 0.98, 3.75 | 500, 500, 986 | Discovery 690 | GE MEDICAL SYSTEMS |
| Subject #33 | No | January 2013 | 74 | Male | 1 | 1 | 2 | 0.98, 0.98, 3.75 | 500, 500, 1121 | Discovery 690 | GE MEDICAL SYSTEMS |
| Subject #34 | No | October 2012 | 52 | Male | 2 | 1 | 0 | 0.98, 0.98, 3.75 | 500, 500, 1121 | Discovery 690 | GE MEDICAL SYSTEMS |
| Subject #35 | No | November 2013 | 65 | Female | 4 | 1.6 | 2 | 0.98, 0.98, 3.75 | 500, 500, 986 | Discovery 690 | GE MEDICAL SYSTEMS |
| Subject #36 | No | May 2011 | 73 | Male | 2 | 0.8 | 2 | 0.83, 0.83, 5.00 | 427, 427, 380 | LightSpeed16 | GE MEDICAL SYSTEMS |
| Subject #37 | No | June 2009 | 74 | Female | 3 | 0.6 | 1 | 0.98, 0.98, 3.75 | 500, 500, 941 | Discovery STE | GE MEDICAL SYSTEMS |
| Subject #38 | No | September 2010 | 63 | Female | 3 | 0.8 | 1 | 0.98, 0.98, 3.75 | 500, 500, 1121 | Discovery 690 | GE MEDICAL SYSTEMS |
| Subject #39 | No | February 2007 | 65 | Female | 1 | 1.6 | 2 | 0.74, 0.74, 0.63 | 380, 380, 330 | Discovery CT750 HD | GE MEDICAL SYSTEMS |
| Subject #40 | No | March 2010 | 73 | Female | 6 | 0.7 | 2 | 0.63, 0.63, 5.00 | 323, 323, 320 | Brilliance 64 | Philips |
| Subject #41 | No | April 2012 | 69 | Male | 6 | 2.4 | 1 | 0.98, 0.98, 3.75 | 500, 500, 1068 | Discovery STE | GE MEDICAL SYSTEMS |
| Subject #42 | No | March 2012 | 73 | Female | 2 | 1.2 | 1 | 0.98, 0.98, 3.75 | 500, 500, 941 | Discovery STE | GE MEDICAL SYSTEMS |
| Subject #43 | No | May 2013 | 73 | Male | 3 | 2 | 2 | 0.98, 0.98, 3.75 | 500, 500, 1256 | Discovery 690 | GE MEDICAL SYSTEMS |
| Subject #44 | No | July 2009 | 62 | Female | 3 | 1 | 2 | 0.70, 0.70, 5.00 | 360, 360, 295 | Discovery CT750 HD | GE MEDICAL SYSTEMS |
| Subject #45 | No | July 2013 | 65 | Male | 4 | 1.3 | 2 | 0.70, 0.70, 5.00 | 360, 360, 360 | SOMATOM Definition | SIEMENS |
| Subject #46 | No | November 2010 | 67 | Female | 4 | 1.9 | 2 | 0.98, 0.98, 3.75 | 500, 500, 986 | Discovery 690 | GE MEDICAL SYSTEMS |
| Subject #47 | No | April 2011 | 75 | Male | 2 | 1.7 | 1 | 0.98, 0.98, 3.75 | 500, 500, 1068 | Discovery STE | GE MEDICAL SYSTEMS |
| Subject #48 | No | January 2013 | 61 | Female | 1 | 2.2 | 2 | 0.98, 0.98, 3.75 | 500, 500, 986 | Discovery 690 | GE MEDICAL SYSTEMS |
| Subject #49 | No | April 2014 | 79 | Female | 2 | 3.4 | 1 | 0.98, 0.98, 3.75 | 500, 500, 941 | Discovery STE | GE MEDICAL SYSTEMS |
| Subject #50 | Yes | October 2012 | 72 | Male | 1 | 1.1 | 2 | 0.98, 0.98, 3.75 | 500, 500, 1196 | Discovery STE | GE MEDICAL SYSTEMS |
| Subject #51 | No | September 2010 | 65 | Male | 0 | 2.6 | 2 | 0.77, 0.77, 5.00 | 396, 396, 365 | SOMATOM Definition Flash | SIEMENS |
| Subject #52 | No | April 2014 | 67 | Male | 3 | 1.1 | 2 | 0.65, 0.65, 0.60 | 332, 332, 391 | SOMATOM Definition Flash | SIEMENS |
| Subject #53 | No | February 2007 | 65 | Male | 3 | 2.2 | 2 | 0.83, 0.83, 5.00 | 425, 425, 350 | SOMATOM Definition | SIEMENS |
| Subject #54 | No | March 2010 | 66 | Male | 3 | 1.2 | 2 | 0.70, 0.70, 5.00 | 360, 360, 335 | LightSpeed16 | GE MEDICAL SYSTEMS |
| Subject #55 | No | August 2011 | 65 | Male | 2 | 1.2 | 2 | 0.75, 0.75, 0.63 | 384, 384, 405 | LightSpeed VCT | GE MEDICAL SYSTEMS |
| Subject #56 | No | August 2008 | 65 | Male | 1 | 0.9 | 2 | 0.82, 0.82, 0.63 | 422, 422, 310 | Discovery CT750 HD | GE MEDICAL SYSTEMS |
| Subject #57 | No | December 2011 | 86 | Female | 4 | 3.2 | 0 | 0.66, 0.66, 5.00 | 340, 340, 325 | LightSpeed Xtra | GE MEDICAL SYSTEMS |
| Subject #58 | No | June 2013 | 80 | Female | 1 | 1.6 | 2 | 0.70, 0.70, 5.00 | 360, 360, 330 | Discovery CT750 HD | GE MEDICAL SYSTEMS |
| Subject #59 | Yes | May 2013 | 57 | Female | 2 | 1.6 | 2 | 0.98, 0.98, 3.75 | 500, 500, 1121 | Discovery 690 | GE MEDICAL SYSTEMS |
| Subject #60 | No | February 2011 | 70 | Female | 4 | 1.9 | 1 | 0.98, 0.98, 3.75 | 500, 500, 1068 | Discovery STE | GE MEDICAL SYSTEMS |
| Subject #61 | No | March 2014 | 57 | Male | 8 | 1.7 | 1 | 0.98, 0.98, 3.75 | 500, 500, 1068 | Discovery STE | GE MEDICAL SYSTEMS |
| Subject #62 | No | September 2009 | 80 | Male | 5 | 2 | 2 | 0.70, 0.70, 5.00 | 360, 360, 320 | LightSpeed16 | GE MEDICAL SYSTEMS |
| Subject #63 | No | February 2013 | 69 | Male | 0 | 1.6 | 2 | 0.98, 0.98, 3.75 | 500, 500, 1121 | Discovery 690 | GE MEDICAL SYSTEMS |
| Subject #64 | No | October 2010 | 80 | Male | 2 | 2.1 | 2 | 0.98, 0.98, 3.75 | 500, 500, 986 | Discovery 690 | GE MEDICAL SYSTEMS |
| Subject #65 | No | May 2012 | 69 | Male | 5 | 2.3 | 1 | 0.98, 0.98, 3.75 | 500, 500, 1121 | Discovery 690 | GE MEDICAL SYSTEMS |
| Subject #66 | No | March 2012 | 79 | Female | 4 | 1.8 | 2 | 0.98, 0.98, 3.75 | 500, 500, 941 | Discovery STE | GE MEDICAL SYSTEMS |
| Subject #67 | No | September 2013 | 51 | Female | 1 | 1.4 | 2 | 0.61, 0.61, 0.63 | 312, 312, 325 | Discovery CT750 HD | GE MEDICAL SYSTEMS |
| Subject #68 | No | July 2013 | 59 | Male | 3 | 2.3 | 2 | 0.98, 0.98, 3.75 | 500, 500, 1068 | Discovery STE | GE MEDICAL SYSTEMS |
| Subject #69 | No | November 2013 | 66 | Female | 1 | 1.2 | 2 | 0.98, 0.98, 4.00 | 500, 500, 1488 | Biograph 6 | SIEMENS |
| Subject #70 | No | August 2008 | 65 | Male | 3 | 1 | 2 | 0.68, 0.68, 5.00 | 350, 350, 360 | LightSpeed16 | GE MEDICAL SYSTEMS |
| Subject #71 | No | August 2010 | 81 | Female | 2 | 1.9 | 2 | 0.70, 0.70, 1.25 | 360, 360, 306 | LightSpeed16 | GE MEDICAL SYSTEMS |
| Subject #72 | No | October 2013 | 66 | Female | 3 | 2.4 | 2 | 0.78, 0.78, 5.00 | 397, 397, 290 | Discovery CT750 HD | GE MEDICAL SYSTEMS |
| Subject #73 | No | January 2010 | 73 | Male | 1 | 6 | 2 | 0.78, 0.78, 0.63 | 397, 397, 345 | Discovery CT750 HD | GE MEDICAL SYSTEMS |
| Subject #74 | No | January 2013 | 77 | Female | 1 | 1.2 | 2 | 0.70, 0.70, 5.00 | 360, 360, 325 | Discovery CT750 HD | GE MEDICAL SYSTEMS |
| Subject #75 | No | April 2011 | 65 | Male | 5 | 2 | 2 | 0.84, 0.84, 5.00 | 430, 430, 335 | Discovery CT750 HD | GE MEDICAL SYSTEMS |
| Subject #76 | No | December 2009 | 60 | Female | 0 | 1.6 | 2 | 0.57, 0.57, 5.00 | 290, 290, 330 | SOMATOM Definition Flash | SIEMENS |
| Subject #77 | Yes | February 2014 | 65 | Female | 3 | 1.6 | 2 | 0.98, 0.98, 3.75 | 500, 500, 941 | Discovery STE | GE MEDICAL SYSTEMS |
| Subject #78 | No | August 2009 | 83 | Female | 4 | 1.3 | 2 | 0.70, 0.70, 5.00 | 360, 360, 340 | LightSpeed16 | GE MEDICAL SYSTEMS |
| Subject #79 | No | June 2011 | 82 | Male | 4 | 3.1 | 1 | 0.98, 0.98, 3.75 | 500, 500, 1068 | Discovery STE | GE MEDICAL SYSTEMS |
| Subject #80 | Yes | May 2007 | 74 | Female | 3 | 2.8 | 2 | 0.98, 0.98, 3.75 | 500, 500, 963 | Discovery STE | GE MEDICAL SYSTEMS |
| Subject #81 | No | August 2010 | 59 | Female | 2 | 1.3 | 1 | 0.70, 0.70, 1.25 | 360, 360, 351 | Discovery CT750 HD | GE MEDICAL SYSTEMS |
| Subject #82 | No | August 2009 | 66 | Male | 4 | 3.9 | 2 | 0.98, 0.98, 3.75 | 500, 500, 1068 | Discovery STE | GE MEDICAL SYSTEMS |
| Subject #83 | No | July 2013 | 65 | Female | 2 | 1.4 | 2 | 0.59, 0.59, 5.00 | 300, 300, 300 | LightSpeed16 | GE MEDICAL SYSTEMS |

**Table III: SBRT Patient Data.**

| Subject | Local Failure | Date of Procedure | Age | Sex | CCI | Tumor Size (cc) | Rx Dose | Rx Fraction | Smoke HX | CT FOV (mm) | CT Pixel Spacing (mm) | CT Manufacturer | CT Model |
| --- | --- | --- | --- | --- | --- | --- | --- | --- | --- | --- | --- | --- | --- |
| Subject #1 | No | July 2013 | 85 | Male | 5 | 1.5 | 5400 | 3 | 1 | 650, 650, 318 | 1.27, 1.27, 2.00 | SIEMENS / MIM Software | Biograph40 |
| Subject #2 | No | September 2012 | 91 | Male | 4 | 2.4 | 5000 | 5 | 2 | 650, 650, 320 | 1.27, 1.27, 2.50 | GE MEDICAL SYSTEMS | LightSpeed RT |
| Subject #3 | No | January 2012 | 95 | Male | 2 | 1.6 | 5400 | 3 | 2 | 600, 600, 312 | 1.17, 1.17, 3.00 | Philips | Brilliance Big Bore |
| Subject #4 | No | November 2012 | 87 | Male | 4 | 1.5 | 5000 | 5 | 2 | 680, 680, 318 | 1.33, 1.33, 2.00 | SIEMENS / MIM Software | Biograph40 |
| Subject #5 | No | September 2014 | 85 | Female | 3 | 2.8 | 6000 | 8 | 2 | 500, 500, 260 | 0.98, 0.98, 2.50 | GE MEDICAL SYSTEMS | Advantage 4D |
| Subject #6 | No | August 2008 | 78 | Female | 3 | 4 | 4800 | 4 | 2 | 650, 650, 407 | 1.27, 1.27, 2.50 | GE MEDICAL SYSTEMS | LightSpeed RT |
| Subject #7 | No | February 2011 | 82 | Male | 6 | 3.5 | 4800 | 4 | 1 | 500, 500, 397 | 0.98, 0.98, 2.50 | GE MEDICAL SYSTEMS | LightSpeed RT |
| Subject #8 | No | September 2010 | 100 | Male | 1 | 2.4 | 4800 | 4 | 2 | 650, 650, 455 | 1.27, 1.27, 2.50 | GE MEDICAL SYSTEMS | LightSpeed RT |
| Subject #9 | No | September 2014 | 62 | Male | 3 | 1.2 | 5000 | 5 | 2 | 500, 500, 320 | 0.98, 0.98, 2.50 | GE MEDICAL SYSTEMS | Advantage 4D |
| Subject #10 | No | January 2013 | 84 | Male | 3 | 1.2 | 5400 | 3 | 2 | 780, 780, 318 | 1.52, 1.52, 2.00 | SIEMENS / MIM Software | Biograph40 |
| Subject #11 | No | February 2009 | 87 | Male | 5 | 3.5 | 4800 | 4 | 2 | 500, 500, 385 | 0.98, 0.98, 2.50 | GE MEDICAL SYSTEMS | LightSpeed RT |
| Subject #12 | Yes | August 2014 | 77 | Male | 3 | 2 | 6000 | 8 | 2 | 780, 780, 318 | 1.52, 1.52, 3.00 | SIEMENS / MIM Software | Biograph40 |
| Subject #13 | No | November 2013 | 77 | Male | 3 | 1.5 | 6000 | 8 | 2 | 780, 780, 318 | 1.52, 1.52, 2.00 | SIEMENS / MIM Software | Biograph40 |
| Subject #14 | No | September 2013 | 83 | Male | 3 | 2.9 | 5800 | 8 | 2 | 650, 650, 423 | 1.27, 1.27, 3.00 | SIEMENS | Biograph40 |
| Subject #15 | No | May 2014 | 81 | Female | 3 | 3.3 | 2000 | 2 | 1 | 600, 600, 258 | 1.17, 1.17, 3.00 | Philips | Brilliance Big Bore |
| Subject #16 | No | January 2013 | 77 | Male | 3 | 2 | 5000 | 4 | 0 | 780, 780, 318 | 1.52, 1.52, 2.00 | SIEMENS / MIM Software | Biograph40 |
| Subject #17 | No | October 2013 | 85 | Male | 3 | 1.7 | 5400 | 3 | 2 | 780, 780, 318 | 1.52, 1.52, 2.00 | SIEMENS / MIM Software | Biograph40 |
| Subject #18 | No | March 2014 | 91 | Female | 3 | 3.8 | 5000 | 5 | 2 | 600, 600, 291 | 1.17, 1.17, 3.00 | Philips | Brilliance Big Bore |
| Subject #19 | No | March 2014 | 79 | Female | 2 | 1.6 | 5400 | 3 | 1 | 650, 650, 318 | 1.27, 1.27, 2.00 | SIEMENS / MIM Software | Biograph40 |
| Subject #20 | No | March 2014 | 85 | Female | 3 | 0.9 | 5400 | 3 | 2 | 600, 600, 252 | 1.17, 1.17, 3.00 | Philips | Brilliance Big Bore |
| Subject #21 | No | January 2011 | 91 | Female | 6 | 2.6 | 4800 | 4 | 2 | 650, 650, 480 | 1.27, 1.27, 2.50 | GE MEDICAL SYSTEMS | LightSpeed RT |
| Subject #22 | No | May 2011 | 94 | Female | 3 | 2 | 4800 | 4 | 2 | 650, 650, 342 | 1.27, 1.27, 2.50 | GE MEDICAL SYSTEMS | LightSpeed RT |
| Subject #23 | No | May 2010 | 89 | Female | 4 | 4 | 5000 | 5 | 2 | 650, 650, 230 | 1.27, 1.27, 2.50 | GE MEDICAL SYSTEMS | LightSpeed RT |
| Subject #24 | No | February 2014 | 77 | Male | 5 | 2.4 | 5400 | 3 | 2 | 600, 600, 444 | 1.17, 1.17, 3.00 | Philips | Brilliance Big Bore |
| Subject #25 | No | July 2014 | 90 | Male | 4 | 1.5 | 5400 | 3 | 2 | 600, 600, 312 | 1.17, 1.17, 3.00 | Philips | Brilliance Big Bore |
| Subject #26 | No | July 2014 | 90 | Female | 2 | 1.3 | 5000 | 4 | 2 | 600, 600, 231 | 1.17, 1.17, 3.00 | Philips | Brilliance Big Bore |
| Subject #27 | No | March 2011 | 68 | Female | 3 | 4.5 | 5000 | 4 | 2 | 500, 500, 390 | 0.98, 0.98, 2.50 | GE MEDICAL SYSTEMS | LightSpeed RT |
| Subject #28 | Yes | February 2007 | 88 | Female | 4 | 4.5 | 6000 | 5 | 2 | 500, 500, 295 | 0.98, 0.98, 2.50 | GE MEDICAL SYSTEMS | LightSpeed RT |
| Subject #29 | No | March 2010 | 95 | Female | 3 | 1.2 | 4800 | 4 | 2 | 650, 650, 437 | 1.27, 1.27, 2.50 | GE MEDICAL SYSTEMS | LightSpeed RT |
| Subject #30 | No | August 2011 | 93 | Female | 5 | 2.1 | 4800 | 4 | 1 | 650, 650, 370 | 1.27, 1.27, 2.50 | GE MEDICAL SYSTEMS | LightSpeed RT |
| Subject #31 | No | August 2008 | 96 | Female | 5 | 2 | 6000 | 3 | 2 | 500, 500, 365 | 0.98, 0.98, 2.50 | GE MEDICAL SYSTEMS | LightSpeed RT |
| Subject #32 | No | July 2014 | 78 | Male | 5 | 2.9 | 5000 | 4 | 2 | 600, 600, 261 | 1.17, 1.17, 3.00 | Philips | Brilliance Big Bore |
| Subject #33 | No | July 2014 | 81 | Female | 5 | 2.5 | 5000 | 5 | 2 | 600, 600, 306 | 1.17, 1.17, 3.00 | Philips | Brilliance Big Bore |
| Subject #34 | Yes | February 2007 | 74 | Male | 4 | 2.5 | 4800 | 4 | 2 | 600, 600, 402 | 1.17, 1.17, 2.50 | GE MEDICAL SYSTEMS | LightSpeed RT |
| Subject #35 | No | July 2009 | 96 | Female | 2 | 3.5 | 4800 | 4 | 2 | 650, 650, 221 | 1.27, 1.27, 1.25 | GE MEDICAL SYSTEMS | LightSpeed RT |
| Subject #36 | No | July 2013 | 82 | Female | 3 | 3.8 | 5000 | 4 | 2 | 650, 650, 296 | 1.27, 1.27, 2.00 | SIEMENS / MIM Software | Biograph40 |
| Subject #37 | No | October 2013 | 80 | Female | 2 | 1.1 | 5400 | 3 | 2 | 780, 780, 318 | 1.52, 1.52, 2.00 | SIEMENS / MIM Software | Biograph40 |
| Subject #38 | No | January 2010 | 91 | Male | 5 | 2.2 | 4800 | 4 | 2 | 650, 650, 356 | 1.27, 1.27, 1.25 | GE MEDICAL SYSTEMS | LightSpeed RT |
| Subject #39 | No | November 2013 | 76 | Female | 0 | 1.5 | 5000 | 5 | 2 | 780, 780, 375 | 1.52, 1.52, 3.00 | SIEMENS | Biograph40 |
| Subject #40 | No | March 2011 | 74 | Male | 1 | 1.4 | 4800 | 4 | 2 | 650, 650, 465 | 1.27, 1.27, 2.50 | GE MEDICAL SYSTEMS | LightSpeed RT |
| Subject #41 | No | April 2011 | 89 | Female | 4 | 1.7 | 5400 | 3 | 2 | 650, 650, 420 | 1.27, 1.27, 2.50 | GE MEDICAL SYSTEMS | LightSpeed RT |
| Subject #42 | No | April 2014 | 85 | Male | 3 | 2.6 | 5400 | 3 | 2 | 500, 500, 284 | 0.98, 0.98, 2.00 | SIEMENS / MIM Software | Biograph40 |
| Subject #43 | Yes | April 2014 | 98 | Male | 5 | 3.6 | 5000 | 4 | 2 | 600, 600, 249 | 1.17, 1.17, 3.00 | Philips | Brilliance Big Bore |
| Subject #44 | No | December 2013 | 79 | Male | 6 | 1.2 | 5400 | 3 | 2 | 650, 650, 340 | 1.27, 1.27, 2.50 | GE MEDICAL SYSTEMS | LightSpeed RT |
| Subject #45 | No | January 2010 | 73 | Male | 2 | 1.8 | 4400 | 22 | 1 | 500, 500, 505 | 0.98, 0.98, 5 | GE MEDICAL SYSTEMS | LightSpeed RT |
| Subject #46 | No | January 2009 | 96 | Male | 3 | 2.8 | 6000 | 3 | 2 | 650, 650, 322 | 1.27, 1.27, 2.50 | GE MEDICAL SYSTEMS | LightSpeed RT |
| Subject #47 | No | July 2009 | 94 | Male | 4 | 2 | 4800 | 4 | 2 | 500, 500, 475 | 0.98, 0.98, 2.50 | GE MEDICAL SYSTEMS | LightSpeed RT |
| Subject #48 | Yes | February 2014 | 75 | Male | 5 | 3.3 | 5000 | 4 | 2 | 600, 600, 282 | 1.17, 1.17, 3.00 | Philips | Brilliance Big Bore |
| Subject #49 | Yes | May 2008 | 81 | Female | 3 | 1.5 | 4400 | 22 | 1 | 500, 500, 450 | 0.98, 0.98, 2.50 | GE MEDICAL SYSTEMS | LightSpeed RT |
| Subject #50 | No | June 2011 | 79 | Male | 2 | 0.9 | 5400 | 3 | 2 | 650, 650, 475 | 1.27, 1.27, 2.50 | GE MEDICAL SYSTEMS | LightSpeed RT |
| Subject #51 | No | April 2011 | 72 | Female | 2 | 1.3 | 5000 | 5 | 2 | 650, 650, 420 | 1.27, 1.27, 2.50 | GE MEDICAL SYSTEMS | LightSpeed RT |
| Subject #52 | No | March 2011 | 86 | Female | 3 | 2.4 | 4800 | 4 | 2 | 650, 650, 240 | 1.27, 1.27, 2.50 | GE MEDICAL SYSTEMS | LightSpeed RT |
| Subject #53 | No | June 2010 | 94 | Male | 4 | 2.4 | 5400 | 3 | 2 | 650, 650, 472 | 1.27, 1.27, 2.50 | GE MEDICAL SYSTEMS | LightSpeed RT |
| Subject #54 | No | April 2011 | 79 | Male | 4 | 4.7 | 5000 | 5 | 2 | 650, 650, 405 | 1.27, 1.27, 2.50 | GE MEDICAL SYSTEMS | LightSpeed RT |
| Subject #55 | No | October 2013 | 89 | Female | 3 | 2.6 | 5400 | 3 | 0 | 780, 780, 360 | 1.52, 1.52, 3.00 | SIEMENS | Biograph40 |
| Subject #56 | No | October 2013 | 81 | Male | 4 | 1.1 | 6000 | 8 | 2 | 650, 650, 310 | 1.27, 1.27, 2.50 | GE MEDICAL SYSTEMS | LightSpeed RT |
| Subject #57 | No | June 2010 | 90 | Male | 4 | 4.4 | 4000 | 5 | 2 | 650, 650, 375 | 1.27, 1.27, 2.50 | GE MEDICAL SYSTEMS | LightSpeed RT |
| Subject #58 | No | March 2011 | 77 | Male | 4 | 3 | 5000 | 5 | 1 | 650, 650, 477 | 1.27, 1.27, 2.50 | GE MEDICAL SYSTEMS | LightSpeed RT |
| Subject #59 | No | December 2013 | 96 | Male | 2 | 1.9 | 5400 | 3 | 2 | 780, 780, 318 | 1.52, 1.52, 2.00 | SIEMENS / MIM Software | Biograph40 |
| Subject #60 | No | August 2009 | 83 | Female | 4 | 2.9 | 4800 | 4 | 2 | 650, 650, 327 | 1.27, 1.27, 2.50 | GE MEDICAL SYSTEMS | LightSpeed RT |
| Subject #61 | No | June 2014 | 80 | Male | 4 | 3 | 5000 | 4 | 1 | 780, 780, 318 | 1.52, 1.52, 2.00 | SIEMENS / MIM Software | Biograph40 |
| Subject #62 | No | August 2011 | 64 | Male | 3 | 1.8 | 4800 | 4 | 1 | 650, 650, 505 | 1.27, 1.27, 2.50 | GE MEDICAL SYSTEMS | LightSpeed RT |
| Subject #63 | No | July 2010 | 81 | Female | 7 | 2.1 | 4800 | 4 | 2 | 650, 650, 410 | 1.27, 1.27, 2.50 | GE MEDICAL SYSTEMS | LightSpeed RT |
| Subject #64 | No | April 2014 | 79 | Male | 5 | 3.4 | 5400 | 3 | 2 | 750, 750, 318 | 1.46, 1.46, 2.00 | SIEMENS / MIM Software | Biograph40 |
| Subject #65 | No | December 2011 | 67 | Female | 3 | 1.9 | 4800 | 4 | 1 | 650, 650, 455 | 1.27, 1.27, 2.50 | GE MEDICAL SYSTEMS | LightSpeed RT |
| Subject #66 | No | September 2010 | 52 | Male | 5 | 1.5 | 5400 | 3 | 2 | 650, 650, 550 | 1.27, 1.27, 2.50 | GE MEDICAL SYSTEMS | LightSpeed RT |
| Subject #67 | No | April 2010 | 87 | Female | 3 | 2.9 | 4800 | 4 | 2 | 500, 500, 445 | 0.98, 0.98, 2.50 | GE MEDICAL SYSTEMS | LightSpeed RT |
| Subject #68 | Yes | May 2012 | 84 | Male | 2 | 1.6 | 6000 | 30 | 2 | 780, 780, 435 | 1.52, 1.52, 3.00 | SIEMENS | Biograph40 |
| Subject #69 | No | November 2011 | 85 | Female | 4 | 4.5 | 5000 | 4 | 2 | 650, 650, 385 | 1.27, 1.27, 2.50 | GE MEDICAL SYSTEMS | LightSpeed RT |
| Subject #70 | No | August 2014 | 95 | Female | 2 | 3.3 | 5000 | 4 | 2 | 600, 600, 276 | 1.17, 1.17, 3.00 | Philips | Brilliance Big Bore |
| Subject #71 | No | May 2014 | 90 | Female | 5 | 3 | 5400 | 3 | 0 | 780, 780, 300 | 1.52, 1.52, 2.00 | SIEMENS / MIM Software | Biograph40 |
| Subject #72 | No | May 2012 | 91 | Female | 5 | 1.5 | 5400 | 3 | 2 | 650, 650, 300 | 1.27, 1.27, 2.50 | GE MEDICAL SYSTEMS | LightSpeed RT |
| Subject #73 | No | July 2010 | 84 | Male | 4 | 1.7 | 4800 | 4 | 2 | 650, 650, 450 | 1.27, 1.27, 2.50 | GE MEDICAL SYSTEMS | LightSpeed RT |
| Subject #74 | No | May 2008 | 92 | Male | 4 | 1.7 | 4800 | 4 | 1 | 650, 650, 460 | 1.27, 1.27, 2.50 | GE MEDICAL SYSTEMS | LightSpeed RT |
| Subject #75 | No | January 2014 | 75 | Male | 3 | 1.3 | 5400 | 3 | 1 | 780, 780, 318 | 1.52, 1.52, 2.00 | SIEMENS / MIM Software | Biograph40 |
| Subject #76 | No | November 2008 | 85 | Male | 2 | 2.9 | 4600 | 23 | 2 | 600, 600, 420 | 1.17, 1.17, 2.50 | GE MEDICAL SYSTEMS | LightSpeed RT |
| Subject #77 | No | July 2011 | 83 | Male | 5 | 2.3 | 5400 | 3 | 2 | 650, 650, 350 | 1.27, 1.27, 2.50 | GE MEDICAL SYSTEMS | LightSpeed RT |
| Subject #78 | No | February 2011 | 95 | Male | 1 | 1.4 | 5400 | 3 | 2 | 650, 650, 427 | 1.27, 1.27, 2.50 | GE MEDICAL SYSTEMS | LightSpeed RT |
| Subject #79 | Yes | January 2012 | 71 | Female | 5 | 1.7 | 5000 | 5 | 2 | 780, 780, 318 | 1.52, 1.52, 2.00 | SIEMENS / MIM Software | Biograph40 |
| Subject #80 | No | September 2009 | 67 | Female | 8 | 2.7 | 4800 | 4 | 1 | 650, 650, 345 | 1.27, 1.27, 2.50 | GE MEDICAL SYSTEMS | LightSpeed RT |
| Subject #81 | No | May 2011 | 74 | Female | 2 | 1.8 | 4500 | 3 | 2 | 650, 650, 432 | 1.27, 1.27, 2.50 | GE MEDICAL SYSTEMS | LightSpeed RT |
| Subject #82 | Yes | March 2013 | 95 | Male | 6 | 3.4 | 5400 | 3 | 2 | 780, 780, 316 | 1.52, 1.52, 2.00 | SIEMENS / MIM Software | Biograph40 |
| Subject #83 | No | March 2007 | 77 | Male | 5 | 2.4 | 6000 | 3 | 1 | 500, 500, 407 | 0.98, 0.98, 2.50 | GE MEDICAL SYSTEMS | LightSpeed RT |
| Subject #84 | No | October 2013 | 93 | Female | 3 | 2 | 5000 | 4 | 2 | 650, 650, 278 | 1.27, 1.27, 2.00 | SIEMENS / MIM Software | Biograph40 |
